# Therapeutic targeting of YOD1 disrupts the PAX-FOXO1/N-Myc feedback loop in rhabdomyosarcoma

**DOI:** 10.1172/jci.insight.193221

**Published:** 2025-12-16

**Authors:** Wenwen Ying, Jiayi Yu, Xiaomin Wang, Jiayi Liu, Boyu Deng, Xuejing Shao, Jinhu Wang, Ting Tao, Ji Cao, Qiaojun He, Bo Yang, Yifan Chen, Meidan Ying

**Affiliations:** 1 Institute of Pharmacology and Toxicology, Zhejiang Province Key Laboratory of Anti-Cancer Drug Research, College of Pharmaceutical Sciences, and; 2 The Innovation Institute for Artificial Intelligence in Medicine, Zhejiang University, Hangzhou, China.; 3 School of Pharmacy, Hangzhou Medical College, Hangzhou, Zhejiang, China.; 4 Nanhu Brain-computer Interface Institute, Hangzhou, China.; 5 Department of Surgical Oncology, Children’s Hospital, Zhejiang University School of Medicine, National Clinical Research Center for Child Health, Hangzhou, China.; 6 School of Medicine, Hangzhou City University, Hangzhou, Zhejiang, China.

**Keywords:** Cell biology, Oncology, Pharmacology, Ubiquitin-proteosome system

## Abstract

Fusion-positive rhabdomyosarcoma (FP-RMS), driven by PAX-FOXO1 fusion oncoproteins, represents the subtype of RMS with the poorest prognosis. However, the oncogenic mechanisms and therapeutic strategies of PAX-FOXO1 remain incompletely understood. Here, we discovered that N-Myc, in addition to being a classic downstream target of PAX-FOXO1, can also activate its expression and form a transcriptional complex with PAX-FOXO1, thereby markedly amplifying oncogenic signaling. The reciprocal transcriptional activation of PAX3-FOXO1 and N-Myc is critical for FP-RMS malignancy. We further identified YOD1 as a deubiquitinating enzyme that stabilizes both PAX-FOXO1 and N-Myc. Knocking down YOD1 or inhibiting it with G5 could suppress FP-RMS growth both in vitro and in vivo, through promoting the degradation of both PAX-FOXO1 and N-Myc. Collectively, our results identify that YOD1 promotes RMS progression by regulating the PAX3-FOXO1/N-Myc positive feedback loop, and highlight YOD1 inhibition as a promising therapeutic strategy that concurrently reduces the levels of both oncogenic proteins.

## Abstract 2

Researchers find PAX-FOXO1 and N-Myc reciprocal regulation amplifies oncogenic signaling in FP-RMS, and blocking YOD1 with G5 could be a new treatment approach.

## Introduction

Rhabdomyosarcoma (RMS), the most common soft tissue sarcoma in children, displays aggressive biological behavior and is associated with unfavorable clinical outcomes. The alveolar subtype (ARMS) in particular carries a poor prognosis (1–3). Approximately 85% of ARMS cases are characterized by chromosomal translocations that generate PAX-FOXO1 fusion oncoproteins, primarily PAX3-FOXO1 and PAX7-FOXO1 (4). These chimeric transcription factors act as master regulators of RMS pathogenesis, driving tumor progression through widespread transcriptional reprogramming (5). The persistent oncogenic activity of PAX-FOXO1 fusion proteins also contributes to therapeutic resistance, rendering patients with fusion-positive RMS (FP-RMS) refractory to conventional multimodality therapies that combine surgery, radiation, and intensive chemotherapy (3, 6). Current risk-adapted management for FP-RMS relies primarily on a multimodal strategy involving (a) core chemotherapy with vincristine, actinomycin D, and cyclophosphamide (VAC); and (b) local control via surgery and radiotherapy. Despite these intensive efforts, no clinically proven targeted therapies are available, and the 5-year survival rate for patients with high-risk disease remains dismal at approximately 30% (7, 8). Although considerable progress has been made in delineating the downstream effector networks controlled by PAX-FOXO1, key gaps persist in our understanding of the upstream regulatory mechanisms that govern the stability and transcriptional activity of these fusion oncoproteins. This incomplete knowledge of the PAX-FOXO1 regulatory axis continues to impede the development of effective molecularly targeted therapies for this high-risk patient population.

N-Myc, a key downstream effector of PAX-FOXO1, is frequently amplified or overexpressed in RMS and contributes to early tumorigenesis in concert with the fusion oncoprotein (9–11). Clinically, aberrant N-Myc activation is observed in approximately 76% of FP-RMS cases, with *MYCN* gene amplification detected in 10%–20% of these tumors (12–14). Moreover, elevated N-Myc protein expression is strongly associated with an unfavorable prognosis, underscoring its clinical relevance as both a potential therapeutic target and a biomarker for risk stratification in FP-RMS. These observations collectively suggest that targeting PAX-FOXO1 alone may be insufficient to fully abrogate tumor progression and point to a more complex regulatory interplay between N-Myc and PAX-FOXO1 than previously appreciated. Further elucidation of the functional relationship between N-Myc and PAX-FOXO1 and their coordinated roles in FP-RMS pathogenesis may therefore provide critical insights for developing effective therapeutic strategies against this aggressive disease.

Targeting oncoproteins for degradation represents a promising strategy in cancer therapeutics. In FP-RMS, the PAX3-FOXO1 fusion exhibits enhanced stability compared with its wild-type counterparts, implicating this property in its oncogenicity and revealing a potential therapeutic window (15, 16). As direct inhibition of transcription factors like PAX-FOXO1 and N-Myc remains a major pharmacological challenge, investigating alternative approaches to perturb their protein stability offers a rational strategy for abrogating their oncogenic functions.

Our study delineates a critical reciprocal regulatory circuit between PAX-FOXO1 and N-Myc that drives transcriptional reprogramming and oncogenesis in RMS. We demonstrate that this interplay profoundly reshapes the transcriptional landscape, activating a malignant gene expression program. Furthermore, we identify the deubiquitinase (DUB) YOD1 as a key regulator essential for stabilizing both oncoproteins, thereby maintaining the aggressive phenotype of RMS cells. Importantly, pharmacological inhibition of YOD1 suppresses tumor growth in patient-derived xenograft models. Our results thus unveil a regulatory layer controlling oncoprotein stability in RMS and position YOD1 as a compelling therapeutic node whose targeting could simultaneously neutralize 2 key drivers of this disease.

## Results

### Reciprocal transcriptional activation of PAX3-FOXO1 and N-Myc in the malignant progression of RMS.

**Figure 1:**
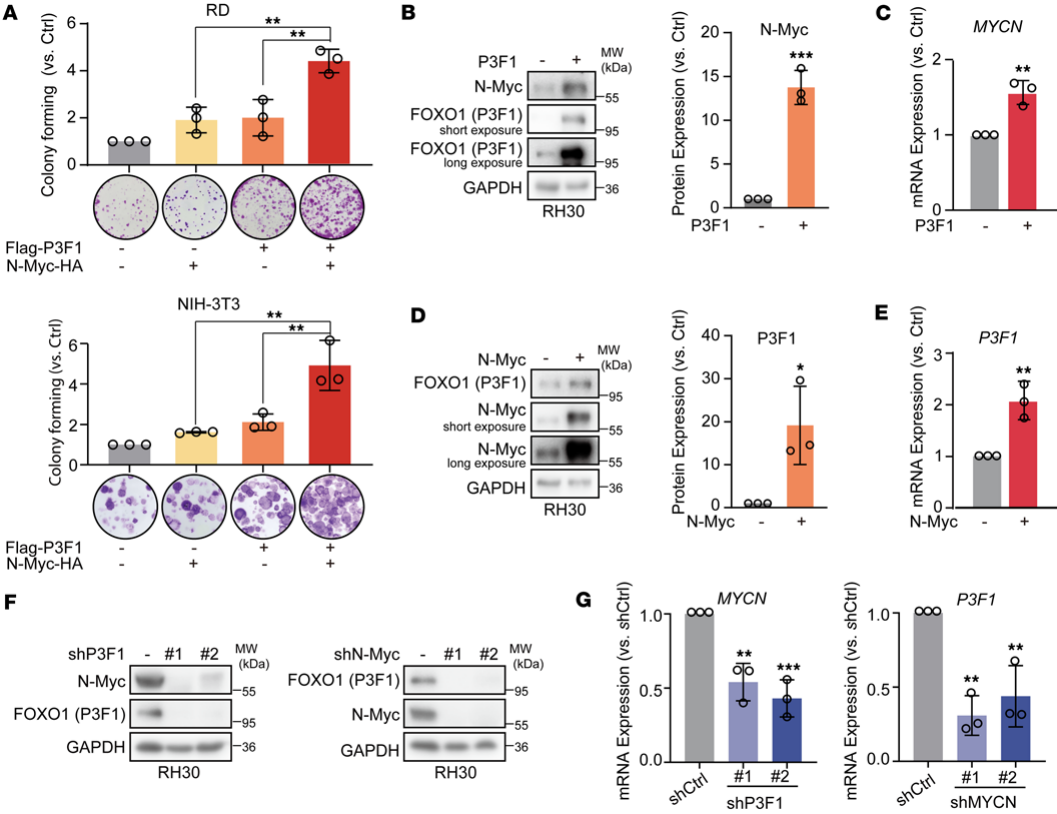
Reciprocal transcriptional activation of PAX3-FOXO1 and N-Myc in the malignant progression of RMS. (**A**) Colony formation assay (SRB) of RD and NIH-3T3 cells transduced with lentiviruses expressing the indicated constructs: empty vector, N-Myc alone, PAX3-FOXO1 (P3F1) alone, or N-Myc and P3F1 together. The bar graph shows the colony formation rate, presented as the ratio of colonies in each overexpression group to the empty vector control. Data represent the mean ± SD. *n* = 3. **P < 0.01 by 1-way ANOVA with Tukey’s multiple-comparison test. (**B**) Overexpression of P3F1 in RH30 cells and its effect on N-Myc protein levels, as determined by Western blot. (**C**) Overexpression of P3F1 in RH30 cells and its effect on *MYCN* mRNA levels, as assessed by RT-qPCR. (**D**) Overexpression of N-Myc in RH30 cells and its effect on P3F1 protein levels, as determined by Western blot. (**E**) Overexpression of N-Myc in RH30 cells and its effect on *P3F1* mRNA levels, as assessed by RT-qPCR. (**B**–**E**) Quantitative analysis of the P3F1/GAPDH or N-Myc/GAPDH protein ratio from multiple independent experiments. Data are presented as mean ± SD. *n* = 3. *P < 0.05, **P < 0.01, ***P < 0.001 by 2-tailed Student’s *t* test. (**F**) RH30 cells were infected with lentiviruses expressing shP3F1 or shMYCN, and the protein levels of N-Myc and PAX3-FOXO1 were analyzed by Western blot. Data are representative of 3 independent experiments. (**G**) RH30 cells were infected with lentiviruses expressing shP3F1 or shMYCN, and the mRNA levels of *P3F1* and *MYCN* were analyzed by qRT-PCR. Data represent the mean ± SD. *n* = 3. **P < 0.01, ***P < 0.001 by 1-way ANOVA with Dunnett’s multiple-comparison test.

To investigate the contribution of N-Myc to FP-RMS pathogenesis, we established stably expressing experimental models in fusion-negative RMS (FN-RMS) RD cells and NIH-3T3 mouse embryonic fibroblasts via lentiviral transduction (Supplemental Figure 1A; supplemental material available online with this article; https://doi.org/10.1172/jci.insight.193221DS1). Initial characterization revealed that N-Myc or PAX3-FOXO1 overexpression alone slightly increased the clonogenic capacity of either cell line. However, coexpression of N-Myc with PAX3-FOXO1 significantly enhanced the clonogenic ability driven by PAX3-FOXO1, increasing colony formation by an average of 2.18-fold in RD cells and 2.32-fold in NIH-3T3 cells, relative to the group expressing only PAX3-FOXO1 (Figure 1A). Surprisingly, in the pooled polyclonal populations, the cotransfection of PAX3-FOXO1 and N-Myc led to higher protein levels of each other compared with their individual transfection (Supplemental Figure 1B), suggesting a potential reciprocal regulatory relationship between these 2 proteins.

To examine whether PAX3-FOXO1 and N-Myc can mutually regulate each other, we first overexpressed PAX3-FOXO1 in RH30 cells and observed a consequent increase in both the protein and mRNA levels of endogenous N-Myc (Figure 1, B and C). Furthermore, we overexpressed N-Myc and found that it similarly enhanced the expression of PAX3-FOXO1 at both the protein and transcript levels (Figure 1, D and E). These findings suggest that N-Myc acts not only as a downstream effector but also as an upstream transcriptional regulator of PAX3-FOXO1, indicating that the 2 proteins can mutually regulate each other to form a positive feedback loop.

To establish the existence of a mutual regulatory circuit, we also individually knocked down *PAX3-FOXO1* and *MYCN* in RH30 cells and then detected the protein levels and mRNA levels of both genes. The results showed that knockdown of either PAX3-FOXO1 or N-Myc strongly reduced the protein (Figure 1F) and mRNA (Figure 1G) levels of the other. These reciprocal effects, observed upon the perturbation of either oncogene, demonstrate that PAX3-FOXO1 and N-Myc engage in a positive feedback loop, validating their transcriptional interdependence and underscoring their potential critical role in RMS pathogenesis.

### PAX-FOXO1/N-Myc positive feedback loop drives global transcriptional reprogramming in RMS.

**Figure 2:**
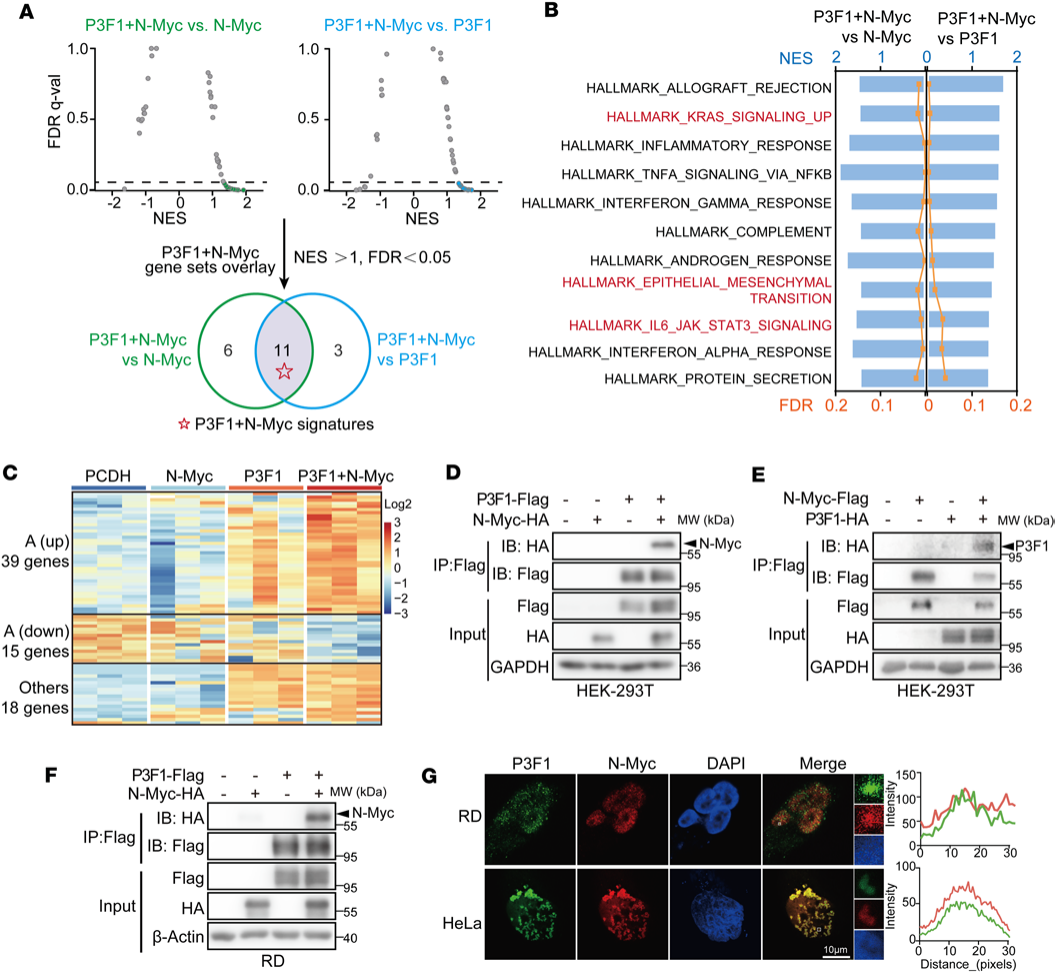
PAX-FOXO1/N-Myc positive feedback loop drives global transcriptional reprogramming in RMS. (**A**) Identification of a Hallmark gene signature coregulated by PAX3-FOXO1+N-Myc. GSEA was performed on transcriptomic data from RD cells comparing the PAX3-FOXO1+N-Myc group versus N-Myc– or P3F1-alone groups. Significantly enriched Hallmark pathways (FDR < 0.05, |NES| > 1) from both comparisons are displayed in the scatter plot. NES, normalized enrichment score. The coregulated signature was defined as the intersection of significant pathways from the 2 comparisons. *n* = 3. (**B**) Combined bar and line graph representing the 11 overlapping Hallmark pathways. Bars show the NES, and the line shows the –log_10_(FDR) for each pathway. (**C**) Heatmap displaying gene expression clusters. Cluster A denotes genes specifically activated in the PAX3-FOXO1+N-Myc group. *n* = 3. (**D**) Immunoprecipitations (IP) by anti-FLAG beads from HEK-293T cells transfected with vector or N-Myc-HA along with vector or P3F1-FLAG for 48 hours were analyzed by immunoblot (IB). (**E**) IPs by anti-FLAG beads from HEK-293T cells transfected with vector or P3F1-HA along with vector or N-Myc-Flag for 48 hours were analyzed by IB. (**F**) IPs by anti-FLAG beads from RD cells transfected with vector or N-Myc-HA along with vector or P3F1-FLAG for 48 hours were analyzed by IB. Data are representative of 3 independent experiments. (**G**) Immunofluorescence analysis showing subcellular colocalization of P3F1 (green) and N-Myc (red) in HeLa and RD cells. HeLa was used as a tool cell. Cells were infected with GFP-P3F1 and N-Myc-HA for 48 hours. Nuclei were stained with DAPI (blue). Colocalization was analyzed using ImageJ. Scale bar: 10 μm.

Based on the reciprocal regulation between PAX3-FOXO1 and N-Myc, we next investigated the global transcriptional changes in this interaction. We generated RD cell models expressing N-Myc alone, PAX3-FOXO1 alone, or both genes simultaneously, with an empty vector as a negative control. In these models, we confirmed that coexpression of both genes induced an enhanced proclonogenic phenotype, as we previously observed. We then analyzed these cells by RNA sequencing (RNA-seq). The results demonstrated that PAX3-FOXO1 overexpression effectively activated its downstream targets by examining the enrichment of a defined PAX3-FOXO1 gene set, confirming the successful establishment of the model (Supplemental Figure 2A). In contrast, N-Myc alone only partially activated a subset of its target genes, which is consistent with the lower viral titer used for its transduction. We then performed gene set enrichment analysis (GSEA) to examine how the transcriptome of the PAX3-FOXO1 and N-Myc coexpression group changed compared with the single-gene expression groups. As shown in Figure 2A, the coexpression group exhibited enrichment of 17 Hallmark pathways compared with N-Myc alone and 14 pathways compared with PAX3-FOXO1 alone. A Venn intersection of these 2 sets revealed 11 overlapped pathways, suggesting they are uniquely activated by the coexpression (Figure 2A). These included cancer-associated pathways such as KRAS signaling, EMT, and JAK/STAT signaling (Figure 2B). To further identify the specific genes coregulated by PAX3-FOXO1 and N-Myc, we analyzed differentially expressed genes (DEGs) in the coexpression group versus each single-expression group, using the empty vector control as baseline. The number of DEGs in the PAX3-FOXO1+N-Myc group far exceeded that in either single-expression group. Among the upregulated genes, only 23.2% (13 of 56) overlapped with the PAX3-FOXO1–alone group and 7.1% (4 of 56) with the N-Myc–alone group (Supplemental Figure 2B), indicating that the majority of gene activation is unique to the coexpression condition. This distinct transcriptional signature was further visualized by heatmap analysis (Figure 2C and Supplemental Table 1). Together, these transcriptomic profiles suggest that coexpression of PAX3-FOXO1 and N-Myc not only regulates the classical downstream targets of each gene but also activates many other tumor-related pathways.

To elucidate the mechanism underlying the extensive transcriptional reprogramming driven by PAX3-FOXO1 and N-Myc coexpression, we investigated their potential physical and functional interplay. Coimmunoprecipitation (co-IP) assays in different cell models confirmed a direct protein-protein interaction between PAX3-FOXO1 and N-Myc (Figure 2, D–F), an association that was also observed for the PAX7-FOXO1 fusion (Supplemental Figure 2C). Given that N-Myc is known to engage with a multitude of protein partners (17), we sought to delineate the interaction domain. Notably, N-Myc remained capable of binding PAX3-FOXO1 even upon truncation of its primary DNA-binding domain (Supplemental Figure 2D), suggesting that their cooperation may operate through a DNA-binding-independent mechanism. This finding led us to hypothesize that the assembly of PAX3-FOXO1 and N-Myc into higher-order biomolecular condensates via phase separation could facilitate their transcriptional synergy, as recently demonstrated for MYC (18). In support of this model, we observed robust colocalization of PAX3-FOXO1 and N-Myc within discrete nuclear condensates (Figure 2G), indicating that phase separation may indeed govern the functional interplay between these 2 oncoproteins. Collectively, our findings position the PAX-FOXO1/N-Myc positive feedback loop as a central driver of global transcriptional reprogramming in RMS, potentially mediated through the formation of phase-separated oncogenic condensates.

### The DUB YOD1 dictates both PAX3-FOXO1 and N-Myc protein stability in RMS.

**Figure 3:**
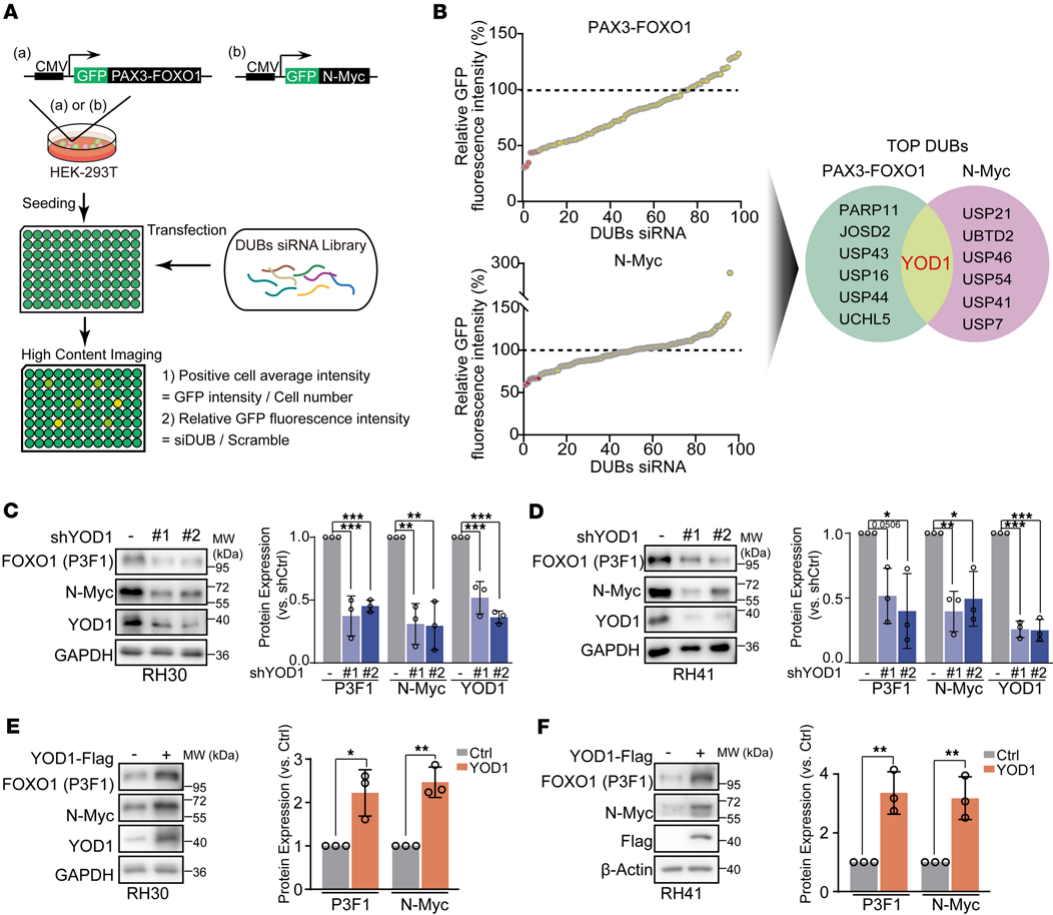
The deubiquitinase YOD1 dictates both PAX3-FOXO1 and N-Myc protein stability in RMS. (**A**) Schematic diagram of the GFP fluorescence reporting system for screening deubiquitinases (DUBs) that regulate the stability of PAX3-FOXO1 and N-Myc. (**B**) Relative fluorescence signal intensity of PAX3-FOXO1 or N-Myc. HEK-293T cells overexpressing GFP-PAX3-FOXO1 or GFP-N-Myc were transfected with siRNAs targeting 98 DUBs. The inhibition rates of the 7 most potent DUB siRNAs were shown to markedly reduce the GFP fluorescence intensity. The Venn diagram displays 7 potential DUB siRNAs with the greatest ability to inhibit the fluorescence intensity of PAX3-FOXO1 and N-Myc effectively. The area of overlap reflects only YOD1 that is present in the 2 protein screening species. (**C** and **D**) Effect of YOD1 knockdown on endogenous P3F1 and N-Myc protein levels. RH30 and RH41 cells were infected with shYOD1 lentivirus and protein levels were assessed by Western blotting. Quantitative analysis of the P3F1/GAPDH or N-Myc/GAPDH protein ratio from multiple independent experiments. Data represent the mean ± SD. *n* = 3. *P < 0.05, **P < 0.01, ***P < 0.001 by 1-way ANOVA with Dunnett’s multiple-comparison test. (**E** and **F**) The effect of YOD1 on endogenous P3F1 and N-Myc protein levels was determined by Western blotting. Quantitative analysis of the P3F1/GAPDH or N-Myc/GAPDH protein ratio from multiple independent experiments. Data are presented as mean ± SD; *n* = 3. *P < 0.05, **P < 0.01 by 2-tailed Student’s *t* test.

The reciprocal regulation between PAX3-FOXO1 and N-Myc, which activates many pro-oncogenic signals and further enhances the malignant phenotype of RMS, underscores the importance of simultaneously targeting both proteins in RMS therapy. Given the challenges in directly inhibiting transcription factors pharmacologically, we adopted an alternative approach focused on modulating their protein stability through the ubiquitin-proteasome system. DUBs are enzymes that remove ubiquitin from proteins, thereby enhancing their stability by preventing proteasomal degradation (19). To systematically identify the DUBs that regulate the stability of PAX3-FOXO1 and N-Myc, we established stable cell models coexpressing each oncoprotein with an EGFP reporter. We then performed a functional siRNA-based screen targeting 98 known human DUBs, using EGFP fluorescence intensity as a quantitative and real-time proxy for oncoprotein abundance (Figure 3A). In the PAX3-FOXO1 screen, siRNA-mediated knockdown of *YOD1*, *PARP11*, and *JOSD2* led to the reduction in fusion protein levels, each decreasing fluorescence by more than 60%. A parallel screen in N-Myc–expressing cells confirmed the known regulator USP7 (20), thereby validating our experimental system. Cross-referencing the top candidate DUBs from both screens revealed YOD1 as the sole enzyme implicated in stabilizing both PAX3-FOXO1 and N-Myc (Figure 3B), suggesting that YOD1 could be a potential DUB of both PAX3-FOXO1 and N-Myc proteins.

To further explore whether YOD1 could regulate the protein stability of both PAX3-FOXO1 and N-Myc, we knocked down YOD1 in 2 PAX3-FOXO1– and N-Myc–positive RMS cell lines, RH30 and RH41 cells. The results showed that knocking down YOD1 caused a reduction in the protein levels of PAX3-FOXO1 and N-Myc (Figure 3, C and D). Conversely, overexpression of YOD1 in the above 2 cell lines led to an obvious increase in the abundance of both PAX3-FOXO1 and N-Myc (Figure 3, E and F). These results suggest that YOD1 may contribute to the stabilization of both PAX3-FOXO1 and N-Myc, supporting its potential role as a DUB involved in the posttranslational regulation of these 2 oncogenic proteins.

### YOD1 serves as a critical DUB in regulating PAX3-FOXO1 and N-Myc stability.

**Figure 4:**
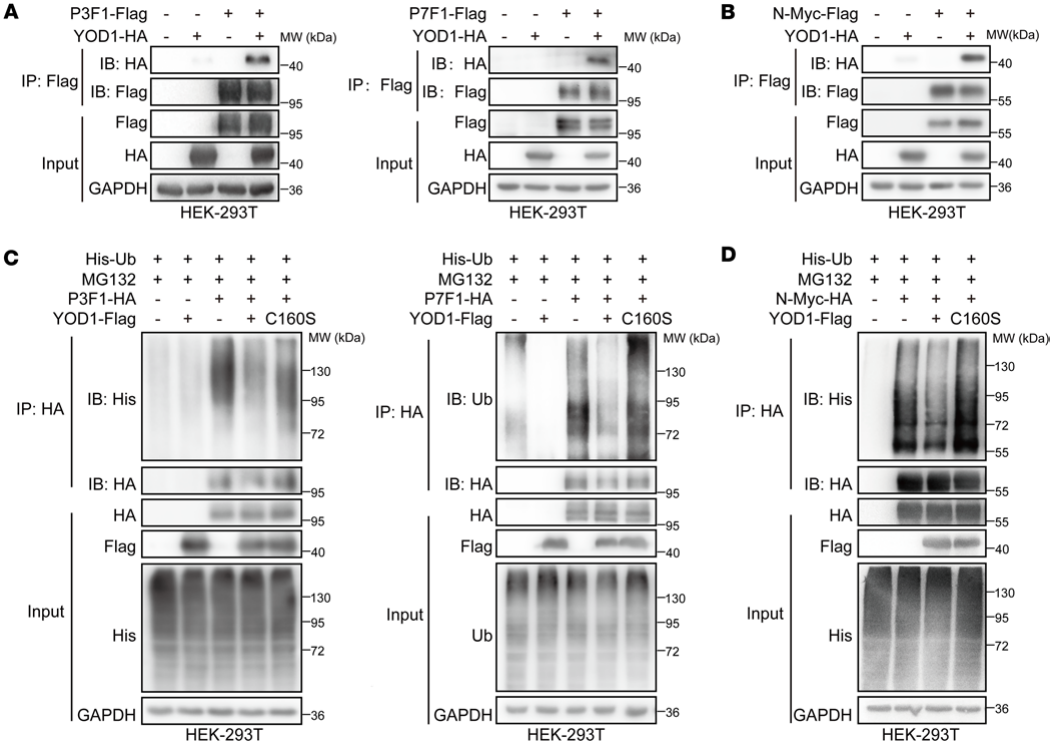
YOD1 serves as a critical DUB in regulating PAX3-FOXO1 and N-Myc stability. (**A** and **B**) The physical interactions between PAX-FOXO1, N-Myc, and YOD1 detected by IP. HEK-293T cells were cotransfected with PAX-FOXO1-FLAG (P3F1 or P7F1) or N-Myc-FLAG and YOD1-HA as indicated. Cell extracts were immunoprecipitated with anti-FLAG beads, and anti-HA antibody was used for detection. (**C** and **D**) The deubiquitinating effect of YOD1 on PAX-FOXO1 and N-Myc in cells were cotransfected with PAX-FOXO1-HA (P3F1 or P7F1) or N-Myc-HA, YOD1-FLAG, and His-Ub as indicated, and then treated with MG132 (8 μmol/L) for 12 hours. Cell extracts were immunoprecipitated with anti-HA beads, and the ubiquitination of PAX-FOXO1 and N-Myc was detected by Western blotting with an anti-His antibody. All data are representative of a minimum of 3 independent repeats. All co-IP experiments were performed by expression of proteins in transiently transfected HEK-293T cells.

YOD1, also known as OTUD2, is a DUB within the OTU family, characterized by its ability to cleave specific ubiquitin chains, thereby modulating protein stability and function (21). To further verify whether YOD1 acts as the DUB for PAX3-FOXO1 and N-Myc, we first examined their interaction by exogenously expressing YOD1 alongside PAX3-FOXO1 and PAX7-FOXO. Our findings demonstrate that overexpressed YOD1 could interact with both PAX3-FOXO1 and PAX7-FOXO1 (Figure 4A). Similarly, a direct biochemical association was confirmed between YOD1 and N-Myc (Figure 4B). These findings collectively establish YOD1 as a binding partner for these key oncogenic transcription factors.

DUBs counterbalance ubiquitin ligase activity by removing ubiquitin chains from substrate proteins, thereby rescuing them from proteasomal degradation. To determine whether YOD1 exhibits deubiquitinating activity toward PAX-FOXO1 and N-Myc, we performed cellular deubiquitination assays. Overexpression of wild-type YOD1 obviously reduced the ubiquitination levels of both PAX3-FOXO1 and PAX7-FOXO1. In contrast, the catalytically inactive YOD1-C160S mutant (22) failed to decrease ubiquitination, underscoring the essential role of YOD1’s enzymatic activity in regulating PAX3-FOXO1 stability (Figure 4C). A consistent effect was observed for N-Myc, where the catalytic mutant also abrogated deubiquitination (Figure 4D). Taken together, these results establish YOD1 as a functional DUB that directly deubiquitinates and stabilizes both PAX3-FOXO1 and PAX7-FOXO1 fusion proteins and N-Myc, highlighting its role as a common regulatory node in the posttranslational control of these oncogenic drivers.

### Depletion of YOD1 inhibits the growth of RMS both in vitro and in vivo.

**Figure 5:**
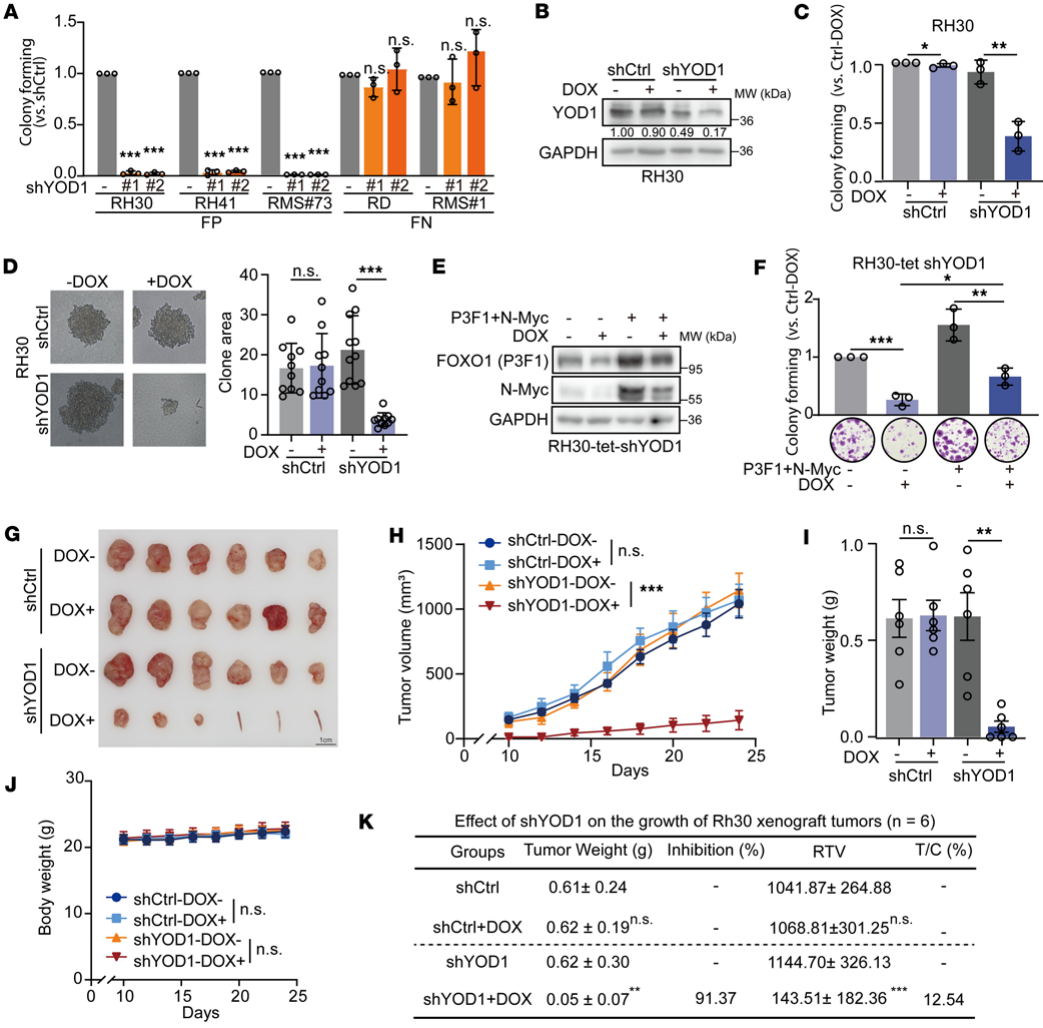
Depletion of YOD1 inhibits the growth of RMS both in vitro and in vivo. (**A**) Colony formation assay in FP-RMS (RH30, RH41) and FN-RMS (RD) cell lines, along with patient-derived cells RMS 73 (FP-RMS) and RMS 1 (FN-RMS), after YOD1 knockdown. Data represent the mean ± SD. *n* = 3. (**B**) Western blot confirming YOD1 knockdown in RH30 Tet-On shYOD1 cells with or without doxycycline (DOX, 2 μg/mL). (**C** and **D**) Clonogenic ability of RH30 Tet-On shYOD1 cells assessed by SRB (**C**) and soft agar assays (**D**). Colony numbers quantified with ImageJ. (**E**) Western blot analysis verifying the exogenous expression of PAX3-FOXO1 and N-Myc in the RH30-tet-shYOD1 cell model. (**F**) Colony formation assays assessing the rescue of clonogenic ability. Cells were subjected to the following conditions: overexpression of PAX3-FOXO1 and N-Myc without DOX induction, and overexpression of PAX3-FOXO1 and N-Myc combined with DOX-induced YOD1 knockdown. (**G**) Tet-On shCtrl or Tet-On shYOD1 images of RH30 xenograft tumors with or without DOX feeding (endpoint). The presence of a mouse tail indicates that the tumor did not grow. (**H**) Tumor volume of Tet-On shCtrl or Tet-On shYOD1 with or without DOX feeding tumors of RH30 xenografts (mean ± SEM, *n* = 6). (**I**) Tumor weight of mice in indicated groups (at the endpoint) of RH30 xenografts (means ± SEM, *n* = 6). (**J**) Body weight of mice in indicated groups (mean ± SEM, *n* = 6). (**K**) The inhibition ratio and T/C value of shYOD1 on RH30 xenograft. *P < 0.05; **P < 0.01; ***P < 0.001 by 1-way ANOVA with Dunnett’s multiple-comparison test (**A**), 2-tailed Student’s *t* test (**C**, **D**, **F**, **I**, and **K**), or 2-way ANOVA with Tukey’s multiple-comparison test (**H** and **J**). NS, *P* > 0.05. RTV, relative tumor volume; T/C, treatment/control.

Considering YOD1’s pivotal role in regulating the protein stability of the PAX-FOXO1 fusion and N-Myc, we proceeded to investigate the potential of YOD1 inhibition to counteract the malignant phenotypes of RMS. We first assessed the impact of YOD1 knockdown on clonogenicity across a panel of FP-RMS and FN-RMS cell lines, as well as patient-derived cells (PDCs) (Supplemental Figure 3A). The results demonstrated that YOD1 knockdown strongly impaired colony formation in FP-RMS models, including RH30, RH41, and RMS 73, whereas minimal effects were observed in FN-RMS cells (RD and RMS 1) (Figure 5A and Supplemental Figure 3, B and C). To rule out potential off-target effects or toxicity associated with constitutive shRNA expression, we established a doxycycline-inducible (DOX-inducible) Tet-On shRNA system for conditional YOD1 knockdown in RH30 and RH41 cells. DOX treatment led to efficient and reproducible downregulation of YOD1 protein (Figure 5B and Supplemental Figure 3D). Consistent with the above results, inducible YOD1 depletion suppressed both cellular proliferation and clonogenic potential in these FP-RMS lines (Figure 5, C and D, and Supplemental Figure 3E). To further investigate whether the suppression of clonogenicity following YOD1 knockdown is dependent on PAX3-FOXO1 and N-Myc, we performed a rescue experiment in the RH30-tet-shYOD1 cell model. Specifically, after inducing YOD1 knockdown with DOX, we reintroduced exogenous PAX3-FOXO1 and N-Myc via transient overexpression (Figure 5E). Colony formation assays revealed that restoring the expression of PAX3-FOXO1 and N-Myc significantly attenuated the clonogenic impairment caused by YOD1 depletion (Figure 5F). These results suggest that the anticlonogenic effect of YOD1 inhibition is highly correlated with the downregulation of PAX3-FOXO1 and N-Myc, reinforcing their functional importance in FP-RMS pathogenesis.

To evaluate the therapeutic potential of YOD1 inhibition in FP-RMS in vivo, we established a xenograft model by subcutaneously implanting RH30 cells into nude mice. The mice were then divided into 2 groups: one transfected with a scramble control and the other with shYOD1. Depletion of YOD1 resulted in a profound suppression of tumor growth, achieving an inhibition rate of 91.37% compared with the control group. Importantly, this potent antitumor effect was well tolerated, as evidenced by no major change in mouse body weight throughout the treatment period (Figure 5, G–K). These findings suggest that shYOD1 effectively suppresses FP-RMS tumor growth, thereby underscoring the therapeutic potential of YOD1 itself.

### YOD1 inhibitor G5 can inhibit the malignant phenotypes of FP-RMS cells.

**Figure 6:**
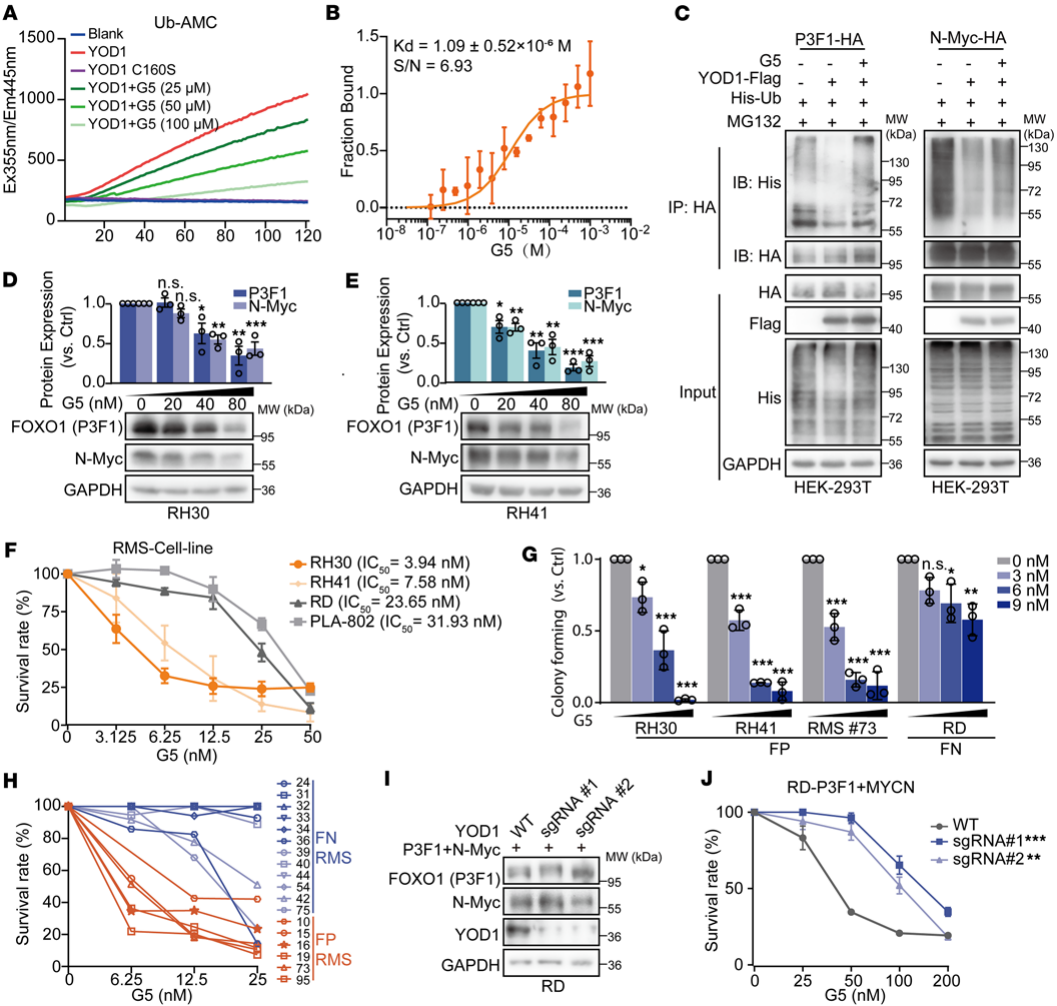
YOD1 inhibitor G5 can inhibit the malignant phenotypes of FP-RMS cells. (**A**) In vitro deubiquitinating activity of GST-YOD1 incubated with G5 (25–100 μM) using Ub-AMC; GST-YOD1-C160S served as negative control. (**B**) Microscale thermophoresis (MST) was used to determine the dissociation constant (*K_d_*) between G5 and YOD1. (**C**) YOD1-mediated deubiquitination of PAX3-FOXO1 (P3F1) and N-Myc in HEK-293T cells cotransfected with His-Ub and YOD1-FLAG, treated with 0.5 μM G5 for 12 hours; IP: HA, IB as shown. Data represent 3 experiments. (**D** and **E**) Western blot and quantification of P3F1 and N-Myc in RH30 and RH41 cells after 24-hour G5 treatment. Protein levels normalized to GAPDH. Data are presented as mean ± SD; *n* = 3. (**F**) IC_50_ of G5 was determined in RH30, RH41, RD, and PLA-802 cell lines following 72-hour treatment. (**G**) RH30, RH41, RMS 73 patient-derived cells (PDCs) and RD cells were seeded in 6-well plates with different concentrations of G5 treatment for 14 days. Data represent the mean ± SD. *n* = 3. (**H**) Dose-response of FP-RMS (*n* = 6) and FN-RMS (*n* = 12) PDCs to G5. Cell viability was measured by CellTiter-Glo assay after 72-hour treatment with the indicated concentrations of G5, and the percentage inhibition of proliferation was calculated. (**I**) Western blot analysis confirming the knockout of YOD1 (using 2 distinct sgRNAs) and subsequent overexpression of PAX3-FOXO1 and N-Myc in RD cells. (**J**) Proliferation inhibition rate in YOD1 wild-type (WT) and knockout (KO) RD cells expressing PAX3-FOXO1 and N-Myc following 72-hour treatment with G5. *n* = 3. *P < 0.05; **P < 0.01; ***P < 0.001 by 2-tailed Student’s *t* test (**D** and **E**), 1-way ANOVA with Dunnett’s multiple-comparison test (**G**), or 2-way ANOVA with Dunnett’s multiple-comparison test (**J**). NS, *P* > 0.05.

Building on our previous finding that G5 can inhibit YOD1’s activity and promote the degradation of PML/RARα (23), we further explored G5’s inhibitory effect on FP-RMS by targeting YOD1 enzymatic activity. We first examined G5’s impact on YOD1’s activity in vitro using a ubiquitin–7-amino-4-methylcoumarin (Ub-AMC) assay. We tested G5’s inhibitory effect at concentrations of 25 μM, 50 μM, and 100 μM on recombinant GST-YOD1, with GST-YOD1-C160S as a negative control. The results showed that G5 could effectively suppress YOD1’s catalytic activity in a dose-dependent manner (Figure 6A). These results indicate that G5 can block YOD1’s enzymatic activity. We then employed microscale thermophoresis (MST) to directly assess the physical interaction between YOD1 and G5. Recombinant human YOD1 protein was fluorescently labeled and titrated with a dilution series of G5. MST analysis revealed a dose-dependent binding curve, with a calculated dissociation constant (*K_d_*) in the 1.09 (±0.52) × 10^–6^ M range (Figure 6B). This robust binding affinity demonstrates a direct and specific molecular interaction between G5 and YOD1. Next, we found that G5 potently rescued the deubiquitylation of PAX3-FOXO1 and N-Myc proteins (Figure 6C). Furthermore, G5 treatment significantly reduced the protein levels of PAX3-FOXO1 and N-Myc in RH30 and RH41 cells (Figure 6, D and E). The simultaneous targeting of the stability of PAX3-FOXO1 and N-Myc suggests that G5 may offer a promising therapeutic approach for diseases characterized by the dysregulation of ubiquitin-dependent processes involving PAX3-FOXO1 and N-Myc.

To evaluate the therapeutic potential of G5 in FP-RMS, we conducted comparative analyses across RMS subtypes: FP-RMS RH30 and RH41 cells (high endogenous N-Myc) versus FN-RMS RD and PLA-802 cells (low endogenous N-Myc) (Supplemental Figure 3A). Strikingly, G5 exhibited much higher inhibitory efficacy in FP-RMS cells (IC_50_ RH30, 3.94 nM; RH41, 7.58 nM) than in FN-RMS models (IC_50_ RD, 23.65 nM; PLA-802, 31.93 nM) (Figure 6F). Moreover, both colony formation and apoptosis assays demonstrated that G5 exerts greater efficacy in FP-RMS compared with FN-RMS (Figure 6G and Supplemental Figure 4A). To further validate the selectivity of G5 in models with higher clinical relevance, we evaluated its antiproliferative effects on a panel of RMS PDC models, encompassing 6 FP- and 12 FN-RMS samples (Supplemental Table 2). The PDCs were treated with a gradient of G5 concentrations, and cell viability was assessed to determine dose-response relationships (Figure 6H). Consistent with the results obtained in RMS cell lines, FP-RMS PDCs exhibited greater sensitivity to G5 treatment compared with their FN-RMS counterparts. These results suggest that G5 may selectively target FP-RMS by downregulating PAX3-FOXO1 and N-Myc, representing a potential therapeutic strategy for FP-RMS.

To establish whether the antitumor effect of G5 on FP-RMS is correlated with YOD1 inhibition, we attempted to generate YOD1-knockout (YOD1-KO) cells in the fusion-positive RH30 background. However, consistent with the essential role of YOD1 in FP-RMS survival, we were unable to obtain viable YOD1-KO RH30 clones, suggesting that complete YOD1 ablation may be lethal in this context. As an alternative strategy, we introduced exogenous PAX3-FOXO1 and N-Myc into a YOD1-KO fusion-negative RD cell line to reconstitute a molecular context resembling FP-RMS (Figure 6I and Supplemental Figure 4B). The results demonstrated that G5 treatment exerted a markedly weaker inhibitory effect on YOD1-KO cells reconstituted with PAX3-FOXO1 and N-Myc compared with their YOD1–wild-type counterparts (Figure 6J). The above results indicate that the efficiency of G5 in FP-RMS is highly correlated with YOD1 inhibition.

### YOD1 inhibitor G5 inhibits FP-RMS tumor growth in vivo.

**Figure 7:**
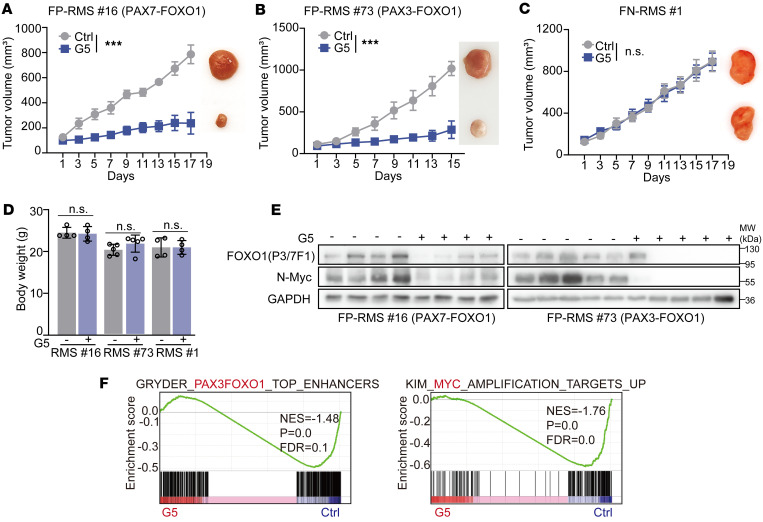
YOD1 inhibitor G5 inhibits FP-RMS tumor growth in vivo. (**A**–**C**) The in vivo effect of G5 on the RMS PDX model (intraperitoneal injections, every other day, 20 mg/kg) for indicated days. Tumor volume of indicated groups was measured every 2 days, mean ± SEM, sample FP-RMS 16, and FN-RMS 1, *n* = 4; FP-RMS 73, *n* = 5). ***P < 0.001 by 2-way ANOVA. NS, *P* > 0.05. (**D**) Body weight of mice in indicated groups (at the endpoint) (mean ± SEM). Differences evaluated by 2-tailed Student’s *t* test. NS, *P* > 0.05. (**E**) Proteins extracted from PDX tumors of FP-RMS. (**F**) RNA-seq GSEA of a tumor isolated from in vivo subcutaneous xenograft model of FP-RMS after treatment with G5 versus control.

To evaluate the in vivo antitumor efficacy of G5 on RMS, we established 2 FP-RMS (RMS 16 and RMS 73) tumor models and 1 FN-RMS (RMS 1) tumor model, and compared their responses to G5 treatment (Supplemental Table 2). The expression levels of *MYCN* and the presence of *PAX-FOXO1* gene fusions in these tissue samples were determined by RNA-seq combined with STAR-Fusion analysis (Supplemental Figure 5A). To ensure the pathological fidelity of these models, we performed transcriptomic sequencing followed by principal component analysis (PCA) on tumor tissues from the patient-derived xenografts (PDXs) and their corresponding patient samples. The analysis revealed a high degree of transcriptional concordance between each PDX model and its original patient tumor, confirming that the PDXs may recapitulate the molecular features of human RMS in vivo (Supplemental Figure 5B). Our results revealed that G5 significant suppressed the tumor growth in mice with FP-RMS–PDX tumors, achieving an inhibition rate of 69.92% in RMS 16 and 72.94% in RMS 73 (Figure 7, A and B). In contrast, G5 had no major effect on the growth of FN-RMS–PDX tumors (Figure 7C). Additionally, G5 treatment did not induce any observable changes in mouse body weights (Figure 7D), indicating that G5 has a favorable selectivity profile, particularly for FP-RMS in vivo.

To determine whether the inhibitory effects of G5 on FP-RMS tumors are attributable to the reduction in PAX3-FOXO1 and N-Myc, we first assessed the protein levels within the above tumors. Our results showed a strong downregulation of PAX3-FOXO1, PAX7-FOXO1, and N-Myc in G5-treated PDX tumors (Figure 7E). In parallel, we conducted RNA-seq analysis on tumor samples obtained from G5-treated mice and control mice, revealing transcriptional profiles altered by G5 treatment (Supplemental Figure 5C). GSEA revealed that G5 treatment downregulated the targets of PAX-FOXO1 and N-Myc, consistent with our in vitro findings (Figure 7F). Additionally, Kyoto Encyclopedia of Genes and Genomes (KEGG) pathway analysis demonstrated that G5 treatment led to the inhibition of various oncogenic signaling pathways and changes in the expression profiles of oncogenes (Supplemental Figure 5D). Notably, within the TGF-β signaling pathway, the expression levels of *ACVR1*, *SMAD1*, *SMAD6*, and *TGIF1* genes, which are implicated in tumorigenesis (24, 25), were obviously modified (Supplemental Figure 5, E and F). These changes inversely correlated with tumor progression, suggesting attenuation of oncogenic processes. Collectively, G5 exhibited potent antitumor activity in FP-RMS PDX models, with therapeutic effects associated with modulation of PAX-FOXO1 and N-Myc protein networks by YOD1 inhibition, supporting further therapeutic potential of the YOD1 inhibition strategy in PAX-FOXO1 FP-RMS.

## Discussion

FP-RMS is a cancer primarily affecting children, characterized by the *PAX-FOXO1* fusion gene, which is a key genetic change and defining feature of the disease (3). Beyond this, additional genetic changes, such as the dysregulation of the *MYCN* oncogene, also contribute to RMS pathogenesis. Our research has revealed a reciprocal transcriptional regulation between PAX-FOXO1 and N-Myc, culminating in the formation of a complex that amplifies oncogenic signals and fuels RMS progression. Crucially, we have identified YOD1, a DUB, as a pivotal coregulator of the stability of both PAX-FOXO1 and N-Myc proteins. The inhibition of YOD1 has been demonstrated to halt RMS growth in both in vitro and in vivo models, positioning YOD1 as a promising therapeutic target for RMS. These discoveries suggest that targeting YOD1 could dismantle the oncogenic PAX-FOXO1–N-Myc complex, presenting a strategy for developing targeted therapies not only for RMS but also for other cancers driven by transcription factor fusions.

While PAX-FOXO1 is recognized as a carcinogenic protein, its expression alone is not sufficient to fully recapitulate RMS in vitro. For example, in certain transgenic animal models, Cre recombinase is required to induce muscle-specific *PAX-FOXO1* expression, and additional mutations in *Ink4a/ARF* and *Trp53* are necessary to promote RMS tumorigenesis (26, 27). Furthermore, PAX-FOXO1 alone cannot fully indicate the prognosis of patients with RMS. The conversion of normal myoblasts into ARMS tumors also requires the overexpression of N-Myc, underscoring the importance of collaborative oncogenes in the oncogenic process (28). The *MYCN* oncogene, frequently upregulated in RMS and considered a downstream effector of PAX-FOXO1, is associated with poor prognosis and plays a pivotal role in tumorigenesis, including cell proliferation, differentiation, and survival (11, 29). While the potential regulatory interplay between N-Myc and PAX3-FOXO1 has been previously suggested, an initial study by Tonelli et al. first highlighted this link (13); subsequent work further noted that PAX3-FOXO1 forms myogenic super-enhancers that collaborate with factors including N-Myc, suggesting an autoregulatory loop (30). Our study further found that YOD1 simultaneously regulates both PAX3-FOXO1 and N-Myc, providing a potential therapeutic target to disrupt the positive feedback loop and thereby suppress PAX-FOXO1 FP-RMS. Notably, YOD1 has been reported to have many other substrates. Our current results demonstrate that PAX3-FOXO1 and N-Myc play important roles in mediating its regulation of RMS malignancy; however, we cannot exclude the possibility that other substrates of YOD1 may also have potential functions, which warrant further investigation. Considering that there are currently no targeted therapies available for FP-RMS, and that PAX3-FOXO1 FP-RMS is often accompanied by high N-Myc expression, PAX3-FOXO1 itself could potentially serve as a biomarker for YOD1 inhibitor therapy, providing a strong rationale for its application in patients with FP-RMS.

Targeting the degradation of fusion oncoproteins by modulating E3 ubiquitin ligases and DUBs is a promising therapeutic strategy for cancer treatment (31, 32). YOD1 has been shown in our previous study to enhance the stability of PML/RARα oncoproteins in acute promyelocytic leukemia (23). In the context of RMS, we identified YOD1 as a regulator of both PAX3-FOXO1 and N-Myc, providing a unique advantage over USP7 (20) by potentially modulating multiple components of the core transcriptional circuitry that drives RMS tumorigenesis. YOD1’s dual regulation suggests a broader impact on the oncogenic process, offering an opportunity for targeted therapies that could disrupt critical oncogenic pathways simultaneously. By inhibiting YOD1, it may be possible to disrupt the positive feedback loop between PAX-FOXO1 and N-Myc, thereby impeding the malignant progression of ARMS. In this study, G5 was utilized primarily as a mechanistic probe to validate our central hypothesis that YOD1 inhibition destabilizes the PAX3-FOXO1–N-Myc complex and suppresses RMS growth. Nonetheless, the development of highly selective YOD1 inhibitors, exemplified by G5, positions them as promising therapeutic candidates for FP-RMS (33–35). Indeed, G5 and related YOD1 inhibitors are under active investigation for their pharmacodynamic profiles and have demonstrated favorable efficacy and safety in preclinical models across multiple cancer types (23, 36). Future work will therefore focus on optimizing this class of compounds and exploring innovative therapeutic strategies.

Our findings enrich the understanding of PAX-FOXO1’s regulatory mechanisms and its oncogenic regulation in RMS. We also underscore the therapeutic potential of targeting the YOD1/PAX3-FOXO1/N-Myc axis in FP-RMS. Further investigation is essential to elucidate the intricate mechanisms underlying YOD1’s function in RMS and to develop YOD1-targeted therapeutics for clinical translation.

## Methods

### Sex as a biological variable.

Sex as a biological variable was not accounted for in this study, as all experiments were conducted exclusively in female mice. The decision to use female mice was based on their relatively docile behavior, which facilitates experimental procedures. The results are expected to be relevant to more than one sex. Sex-specific effects were not evaluated in this study.

### Cell and culture.

RMS cell lines RH30 (RRID: CVCL_0041), RH41 (RRID: CVCL_2176), RD (RRID: CVCL_1649), and PLA-802 (RRID: CVCL_AX34) were provided in-house. The NIH-3T3 (catalog 400101/p677_NIH-3T3) cell line was purchased from the Cell Bank of the Chinese Academy of Sciences. HEK-293T (RRID: CVCL_0063) and 293FT (RRID: CVCL_6911) cell lines were supplied by Invitrogen. HeLa (RRID: CVCL_0030) cells were purchased from Procell Life Science Technology Co., Ltd. The RD, PLA-802, HEK-293T, HEK-293FT, NIH-3T3, and HeLa cells were maintained in DMEM. RH30 cells were cultured in RPMI 1640 medium with 2 mM L-glutamine (Invitrogen). All media were supplemented with 10% fetal bovine serum (Gibco) and 1% penicillin/streptomycin. The cell lines were maintained at 37°C in a humidified atmosphere containing 5% CO_2_ and passaged for a maximum of 2 months. All human cell lines were regularly authenticated by STR profiling and frequently tested for mycoplasma contamination.

### Plasmids.

pCDNA3-PAX3-FOXO1/FKHR (Addgene plasmid 115526) was a gift from Martine Roussel (St. Jude Children’s Research Hospital, Memphis, Tennessee, USA). The full-length cDNA sequences of PAX7-FOXO1, N-Myc, and YOD1 were amplified from the total RNA extractions by PCR, then subcloned into pCDNA3.0 or pCDH vectors. YOD1 mutations were generated by site-directed mutagenesis and verified by sequencing. The packaging plasmid pRΔ8.9 and envelope plasmid pCMV-VSV-G (Addgene plasmid 12259) were provided by D.B. Kohn (UCLA, Los Angeles, California, USA). Sequences for gene-specific shRNAs are listed in Supplemental Table 3.

### Antibodies and reagents.

Antibodies against FOXO1 (catalog 2880), N-Myc (catalog 84406), and FLAG (catalog 14793) were purchased from Cell Signaling Technology. Antibodies against YOD1 (catalog 25370-1-AP) were purchased from Proteintech. Antibodies for PAX3 (catalog ab180754) and PAX7 (catalog ab61067) were purchased from Abcam. Antibodies against HA (catalog db2603), β-actin (catalog db10001), and GAPDH (catalog db106 were purchased from Diagbio. The anti-His antibody (catalog R1207-2) was purchased from HuaBio.

G5, MG132, and DOX were purchased from MedChemExpress and dissolved in DMSO. The final solvent concentration in all experiments was ≤0.1% (v/v). The DUB siRNA library was purchased from Dharmacon (Human ON-TARGETplus siRNA Library-Deubiquitinating Enzymes). It uses ON-TARGETplus technology and is provided in the form of a SMARTpool containing targets of 98 genes, for a total of 392 clones. The siRNA size of each target was 0.1 nmol.

### Real-time PCR.

Real-time PCR was used to detect the mRNA levels of PAX3-FOXO1, PAX7-FOXO1, N-Myc, and YOD1. GAPDH was used as an internal standard. The primers used are shown in Supplemental Table 4.

### Lentivirus production and transduction.

The production, titration, and transduction were carried out as previously described (16). In brief, for lentivirus production, the transgene was cloned into a pCDH vector, which was then cotransfected with packaging plasmids into 293FT cells using a standard protocol. The resulting viral supernatant was used to transduce target cells, and stable polyclonal pools were created through puromycin selection.

### DUB siRNA library screening.

HEK-293T cells were infected with EGFP-PAX3-FOXO1 or N-Myc-EGFP lentivirus and then placed into 96-well microplates (Corning). The cells were transfected with DUB siRNAs (Dharmacon) using jetPrime (Polyplus). After 36 hours of culturing, the cells were fixed with 4% paraformaldehyde and stained with DAPI. Subsequently, high-throughput imaging of EGFP and DAPI signals from each cell was performed using an ImageXpress Pico scanner (Molecular Devices). The average EGFP intensity was measured to assess protein expression. The fold change in relative EGFP expression was normalized to that of the scramble control.

### Immunofluorescence.

RD and HeLa cells expressing EGFP-tagged PAX3-FOXO1 or HA-tagged N-Myc were precultured in fluorescence chamber slides until they reached 80% confluence. They were then fixed with precooled 4% paraformaldehyde at room temperature for 15 minutes and washed 3 times with PBS. Cell permeability was maintained at room temperature for 30 minutes using a blocking buffer (0.4% Triton X-100, 2% BSA in PBS). The cells were subsequently incubated with the antibody (diluted in blocking buffer at 1:1000) at 4°C overnight. After washing with PBS 3 times, the cells were incubated with Alexa Fluor 488–coupled anti-rabbit antibodies (diluted 1:1000 in blocking buffer) at room temperature and protected from light for 1 hour. Following 1 wash with PBS, DAPI (diluted in PBS at 1:1000) was incubated with the cells at room temperature for 5 minutes. The slides were sealed with an antiquenching agent, and the images were acquired using confocal microscopy (TCS SP8, Leica).

### Immunoblotting analysis and IP.

For the immunoblotting analysis, cells were lysed in RIPA lysis buffer (50 mM Tris-base, 150 mM NaCl, 1% Triton X-100, 0.1% SDS, and 1% sodium deoxycholate, pH 7.4) supplemented with protease inhibitors leupeptin, PMSF, and Na_3_VO_4_. Protein concentrations in the cell lysates were measured using a BCA Protein Quantification Kit (YEASEN, 20201ES86). The lysates were then boiled for 5 minutes and separated by SDS-PAGE. For the IP assay, cells were lysed either in RIPA lysis buffer directly or in 4% SDS lysis buffer (4% SDS, 150 mM NaCl, 50 mM triethylamine, pH 7.4), followed by sonication. The cell lysate was then incubated with a 10 μL suspension at 4°C overnight. The immunocomplexes were washed 5 times with RIPA lysis buffer and then analyzed by immunoblotting with the indicated antibodies. Representative images from Western blotting and IP analyses are shown, and all findings were consistently replicated in at least 3 independent experiments.

### In vivo deubiquitination assay.

For the in vivo deubiquitination assay, HEK-293T cells were transfected with the indicated plasmids. After 36 hours, cells were treated with MG132 (10 μmol/L) for 8 hours before harvest. To denature the lysates, they were heated at 95°C for 15 minutes in the presence of 4% SDS lysis buffer, followed by a 10-fold dilution with NP40 lysis buffer (1% NP40, 50 mM Tris-base, 150 mM NaCl, and 10% glycerol). The cell lysates were then incubated with anti-HA beads overnight at 4°C. Subsequently, the beads were washed with NP40 lysis buffer 5 times and analyzed by immunoblotting.

### Cell proliferation assay and colony formation assay.

For the cell proliferation assay, we seeded the specified number of cells per well in 96-well plates. Cell number (absorbance) was estimated using the sulforhodamine B (SRB) assay from Sigma-Aldrich, and the absorbance of the individual wells was determined at an optical density of 515 nm. In colony formation assays, a specified number of cells were inoculated into each well of a 6-well plate. After 2 weeks, the cells were stained with SRB. The stained clone was then photographed, and the area of the clone was measured using ImageJ (RRID: SCR_003070).

### Cell apoptosis analysis.

Apoptosis was quantitatively analyzed by flow cytometry using Annexin V–FITC/PI staining. Briefly, intact cells were gated based on forward and side scatter properties to exclude cellular debris. The apoptosis rate was determined by analyzing the percentage of cells in the Annexin V^+^/PI^−^ population (indicating early apoptosis) and the Annexin V^+^/PI^+^ population (representing late apoptosis or necrosis). Quadrant boundaries were established using unstained and single-stained controls for accurate population discrimination.

### RNA-seq assay.

RNA-seq was performed by Shanghai Biotechnology Corporation, using a previously described methodology (37). The analysis included 3 biological replicates per condition to ensure statistical reliability. After generating the gene expression matrix, low-abundance genes were filtered out by removing those with a total count across all samples of less than 10. DEGs were identified using the DESeq2 package (38). The resulting *P* values were adjusted for multiple testing via the Benjamini-Hochberg procedure to control the false discovery rate (FDR), generating adjusted *P* values (*q* values). DEGs were defined as genes with a *q* value of 0.1 or less and an absolute fold-change of 1.5 or greater. GSEA was subsequently performed on the filtered gene expression matrix. Gene sets with a normalized enrichment score (NES) of greater than 1 and an FDR of less than 0.05 were considered statistically significant. Additionally, STAR-Fusion software (39) was employed to detect high-confidence fusion transcripts, which required the presence of both junction reads and spanning fragments as supporting evidence.

### Ub-AMC assay.

Ub-AMC was purchased from KS-V Peptide Biotechnology and its purity was 95%. To start the reaction, 800 nM YOD1, 50 μM G5, and buffer (50 mM Tris-HCl [pH 7.4], 1 mM EDTA, 1 mg/mL ovalbumin, 1 mM ATP [freshly prepared], 1 mM MgCl_2_ [freshly prepared], and 1 mM DTT [freshly prepared]) were added to the system in a total volume of 50 μL. The plate was then preincubated on ice for 2 hours, after which 8 μM Ub-AMC was added, and the plate was measured at Ex 355/Em 445 using a multifunctional enzyme marker (TECAN). Fluorescence intensity was recorded every 1 minute for 120 minutes.

### MST assay.

A 20 mM stock solution of G5 was serially diluted in PBS-T across 16 concentrations (20 μL each). The target protein (YOD1) was diluted to 200 nM in PBS-T and mixed with each compound concentration. MST measurements were performed in capillaries at 25°C using medium power and 20% LED intensity. Binding affinity was determined with MO Analysis software (NanoTempter Technologies) by fitting the 20-second thermophoresis data to either a *K_d_* or Hill model, followed by normalization to fraction bound.

### Generation of YOD1-KO cells by CRISPR/Cas9.

The generation of YOD1-KO cells mediated by CRISPR/Cas9 was performed as previously described (40). The sgRNA oligonucleotide sequences and primers for YOD1 PCR are supplied in Supplemental Table 5.

### Animal studies.

BALB/c nude mice (4–5 weeks old) were purchased from Hangzhou Ziyuan Experimental Animal Technology Co., LTD (SYXK; 2020-0028). M-NSG mice (catalog NM-NSG-001, 4–5 weeks old) were purchased from Shanghai Model Organisms Center, Inc (SCXK; 2019-0002). All BALB/c nude mice and M-NSG mice were housed in a specific pathogen–free facility at the Drug Safety Evaluation Research Center, Zhejiang University. For the subcutaneous xenograft model, RH30 Tet-On cells expressing either control shRNA (shCtrl) or YOD1-targeting shRNA (shYOD1) were inoculated into the axillary region of nude mice at a density of 4 × 10^6^ cells per 200 μL of medium. Following injection, the mice were randomly assigned to receive either a standard diet or DOX-containing diet. This dietary regimen was initiated on the day of tumor cell inoculation and maintained throughout the entire study period. When the tumors became palpable, their volume was measured at specific time points using a vernier caliper every other day at consistent times. The tumor volume was calculated using the following formula: 0.5 × length × width^2^. The ethical guidelines established by our institutional review board dictate that the humane endpoint for our animal subjects is reached when an individual tumor’s mass constitutes more than 10% of the animal’s total body weight, or when the mean tumor diameter in adult mice surpasses 20 mm. In this study, we ensured that the maximum tumor burden was not exceeded.

PDX was established in our laboratory with female NSG mice implanted with tumor tissue from surgical patients subcutaneously into the axillary region. Sequencing analysis showed that the PDX model closely resembles the original tumor at the molecular level. Once the tumor volume reached approximately 50 mm^3^, we randomly assigned the mice to either a control group or a group that received G5 treatment. The G5 was administered at a concentration of 20 mg/kg through intraperitoneal injections once every 2 days for 3 weeks.

### Statistics.

All measured parameters were averaged for samples under different experimental conditions, and the SD or SEM was calculated. The statistical significance of differences between groups was determined using an unpaired 2-tailed Student’s *t* test, 1-way ANOVA, or 2-way ANOVA. The results were considered significant when *P* was less than 0.05.

### Study approval.

RMS PDCs were established from fresh tumor tissues obtained from surgical resections of patients with RMS (ZJUCH-RMS cohort), as previously described (41). This study was approved by the Ethics Committee of Children’s Hospital, Zhejiang University School of Medicine (2020-IRB-049). The Animal Research Committee at Zhejiang University approved all animal studies and animal care was provided following the institutional guidelines (IACUC-25-S253).

### Data availability.

All resource data are included in the Supporting Data Values file. The raw sequence data reported in this paper have been deposited in the Genome Sequence Archive (Genomics, Proteomics & Bioinformatics 2025) in National Genomics Data Center (Nucleic Acids Res 2025), China National Center for Bioinformation/Beijing Institute of Genomics, Chinese Academy of Sciences (GSA-Human: HRA014178) that are publicly accessible at https://ngdc.cncb.ac.cn/gsa-human Additional information is available upon request from the corresponding author.

## Author contributions

WY: project administration, investigation, and manuscript writing. JY: validation, methodology. XW: investigation, validation. JL: formal analysis, validation. BD: validation. XS: data curation. JW: provided the RMS cell lines RH30, RH41, RD, and PLA-802. TT: resources. JC: visualization. QH: resources, supervision. BY: resources, supervision. YC: project supervision, writing, funding acquisition. MY: conceptualization, funding acquisition, supervision.

## Funding support

- National Natural Science Foundation of China (grant no. 82272677 to MY).
- Natural Science Fund for Distinguished Young Scholars of Zhejiang Province (no. LR23H310001 to MY).
- Postdoctoral Fellowship Program of CPSF (no. GZC20232321 to YC).
- Fundamental Research Funds for the Central Universities (226-2025-00136 to MY).

## Supplementary Material

Supplemental data

Unedited blot and gel images

Supplemental table 1

Supporting data values

## Version 1 12/16/2025

In-Press Preview

## Version 2 02/09/2026

Electronic publication
