# Supplementary material for: Therapeutic targeting of YOD1 disrupts the PAX-FOXO1/N-Myc feedback loop in rhabdomyosarcoma
Source: JCI Insight. 2025 Dec 16;11(3):e193221. doi: 10.1172/jci.insight.193221 (PMC12892920; doi:10.1172/jci.insight.193221)
Supplement: Supplemental data [file jciinsight-11-193221-s060.pdf]

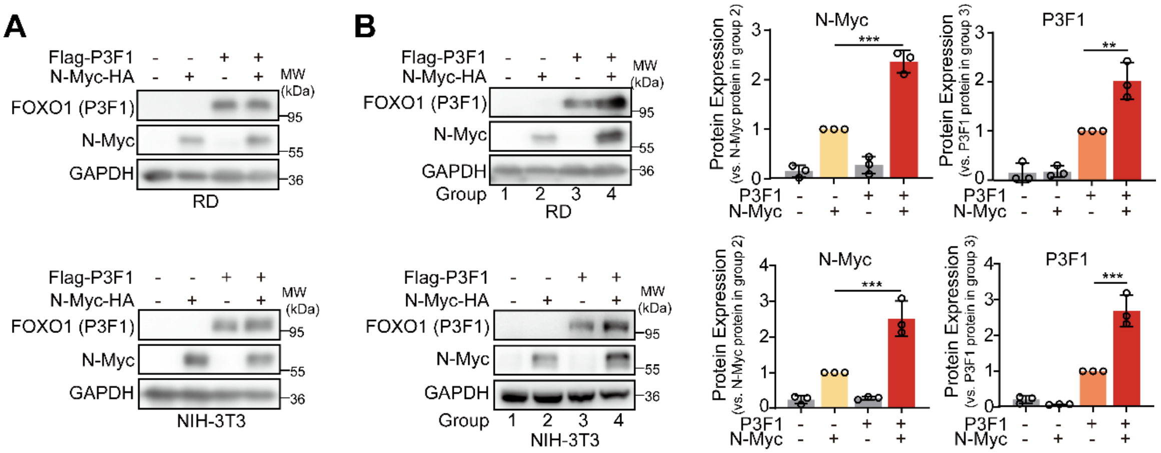

Supplementary Figure 1. Co-expression of PAX3-FOXO1 and N-Myc regulates their mutual expression levels. (A) After lentivirus infection Day3, protein levels of P3F1 and N-Myc were evaluated by Western blotting. **(B)** Western blot analysis of protein lysates from pooled cell colonies (harvested from a single well). Protein expression levels were normalized to the loading control (GAPDH). The bar graph shows the fold change in protein expression in the co-expression group relative to the single overexpression group. Data are presented as mean ± SD ($n = 3$). Data represent the mean ± SD. 1-way ANOVA with Tukey's multiple comparisons test; $^{**}P < 0.01$, $^{***}P < 0.001$.

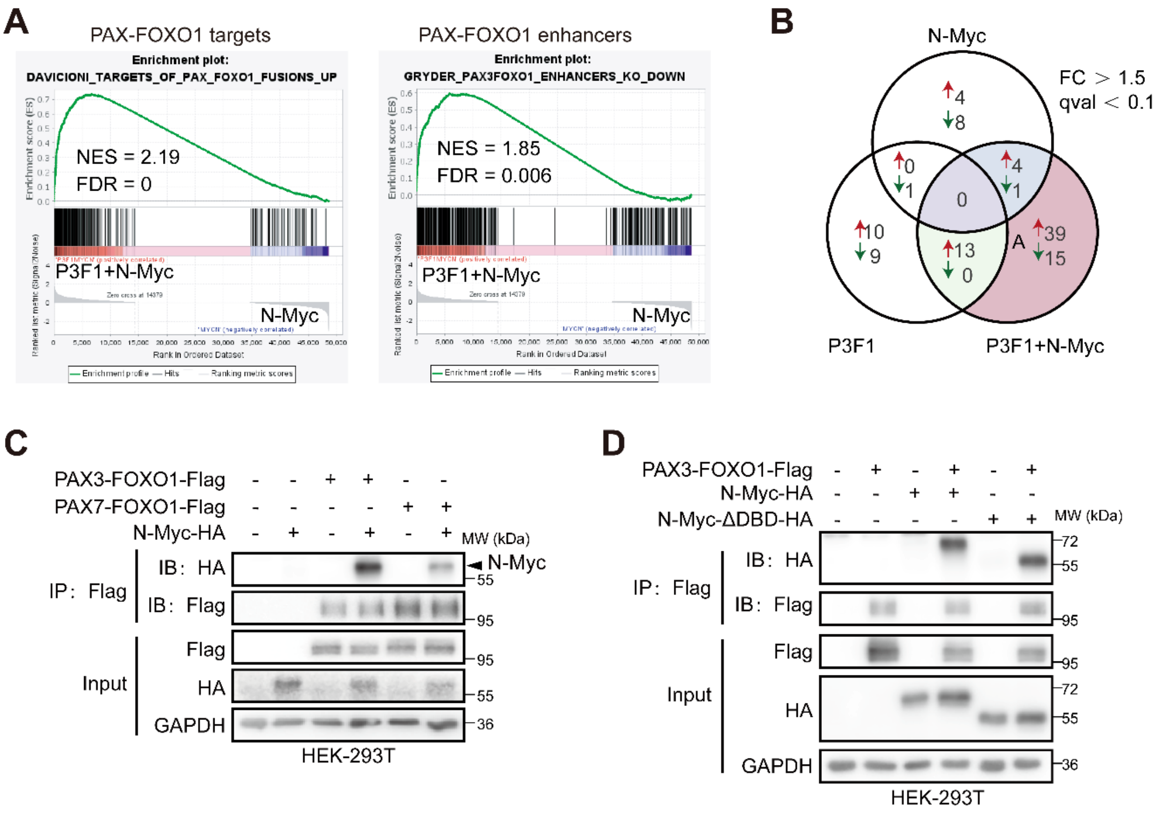

Supplementary Figure 2. Reciprocal Regulation between PAX3-FOXO1 and N-Myc. (**A**) GSEA of transcriptomes from P3F1+N-Myc co-expressing cells versus N-Myc-only cells. Analysis was performed using a defined PAX3-FOXO1 (P3F) target and enhancer gene set to assess pathway enrichment. **(B)** Venn diagram of differentially expressed genes from the comparison of N-Myc, P3F1, and P3F1+N-Myc groups with the empty vector control. **(C)** IPs by Flag beads from HEK-293T cells transfected with vector or N-Myc-HA along with vector, P3F1-Flag or P7F1-Flag for 48 h were subjected to IB. **(D)** IPs by Flag beads from HEK-293T cells transfected with vector, N-Myc-HA or N-Myc-ΔDBD-HA along with vector, P3F1-Flag or P7F1-Flag for 48 h were subjected to IB. Data are representative of three independent experiments.

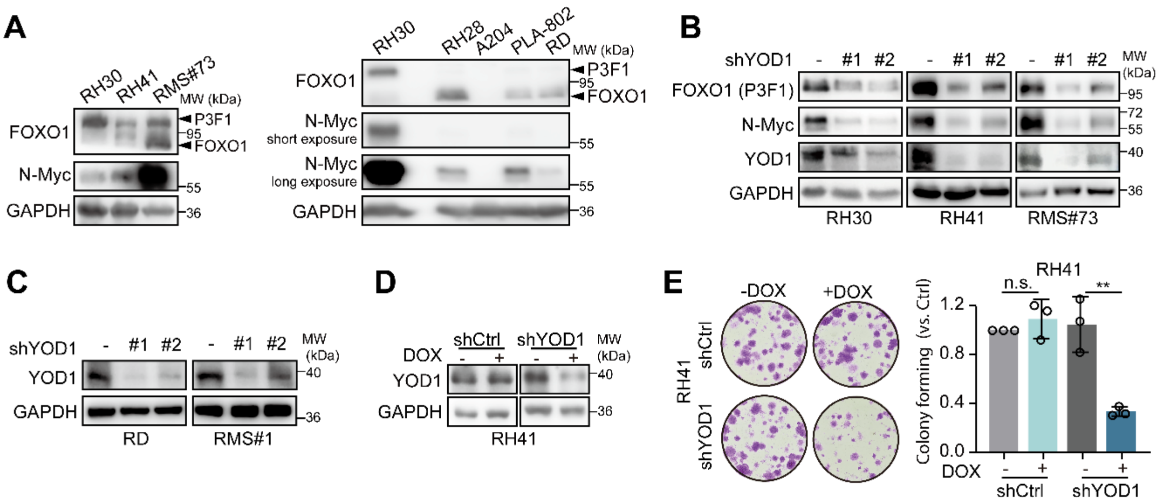

Supplementary Figure 3. YOD1 plays a crucial role in the development of rhabdomyosarcoma. **(A)** Characterization of RMS cell line models. Western blot showing endogenous expression levels of PAX3-FOXO1 and N-Myc, which delineates fusion-positive (FP-RMS) from fusion-negative (FN-RMS) models. **(B)** YOD1 knockdown in FP-RMS models. Western blot analysis of P3F1, N-Myc, and YOD1 protein levels following shRNA-mediated YOD1 knockdown in FP-RMS cell lines (RH30, RH41) and patient-derived cells (PDCs, RMS#73). **(C)** YOD1 knockdown in FN-RMS models. Protein levels of YOD1 was assessed by Western blot after shYOD1 knockdown in FN-RMS cell lines (RD) and PDCs (RMS#1). **(D)** Inducible YOD1 knockdown in RH41 cells. YOD1 expression was analyzed by Western blot with or without doxycycline (DOX, 2 μg/mL) induction. **(E)** Clonogenic assay in RH41 cells with inducible YOD1 knockdown. Colonies were stained with sulforhodamine B (SRB) and quantified using ImageJ. The bar graph represents the fold change in colony formation capacity relative to the shCtrl-DOX group. Student's t test. $n = 3$. n.s: $P > 0.05$, $^{**}P < 0.01$.

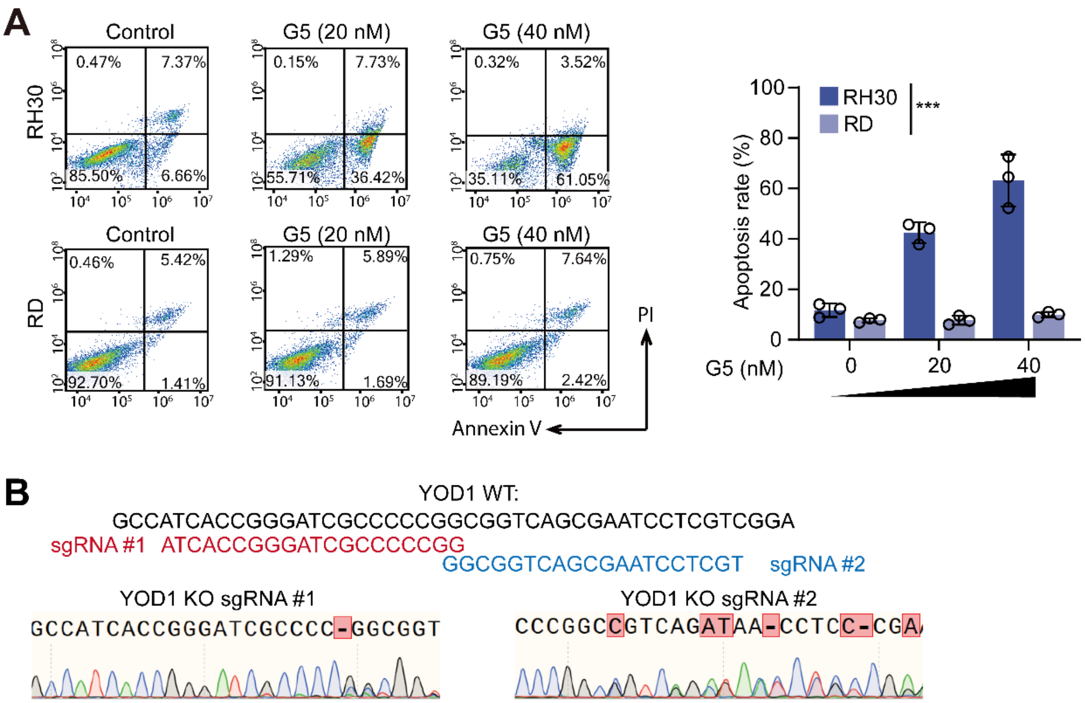

Supplementary Figure 4. G5 exhibits anti-tumor activity in FP-RMS cell. (A) G5 induces a time-dependent apoptotic response. FP-RMS (RH30) and FN-RMS (RD) cells were treated with G5, and the induction of apoptosis was analyzed by flow cytometry following Annexin V and PI staining at the indicated time points. The histogram summarizes the quantitation of total apoptotic cells (mean ± SD, $n = 3$). 2-way ANOVA. ***$P < 0.001$. **(B)** Genotypic confirmation of YOD1 knockout. Sanger sequencing traces of the genomic region targeted by the YOD1-specific sgRNA, confirming the introduction of indels that disrupt the YOD1 coding sequence.

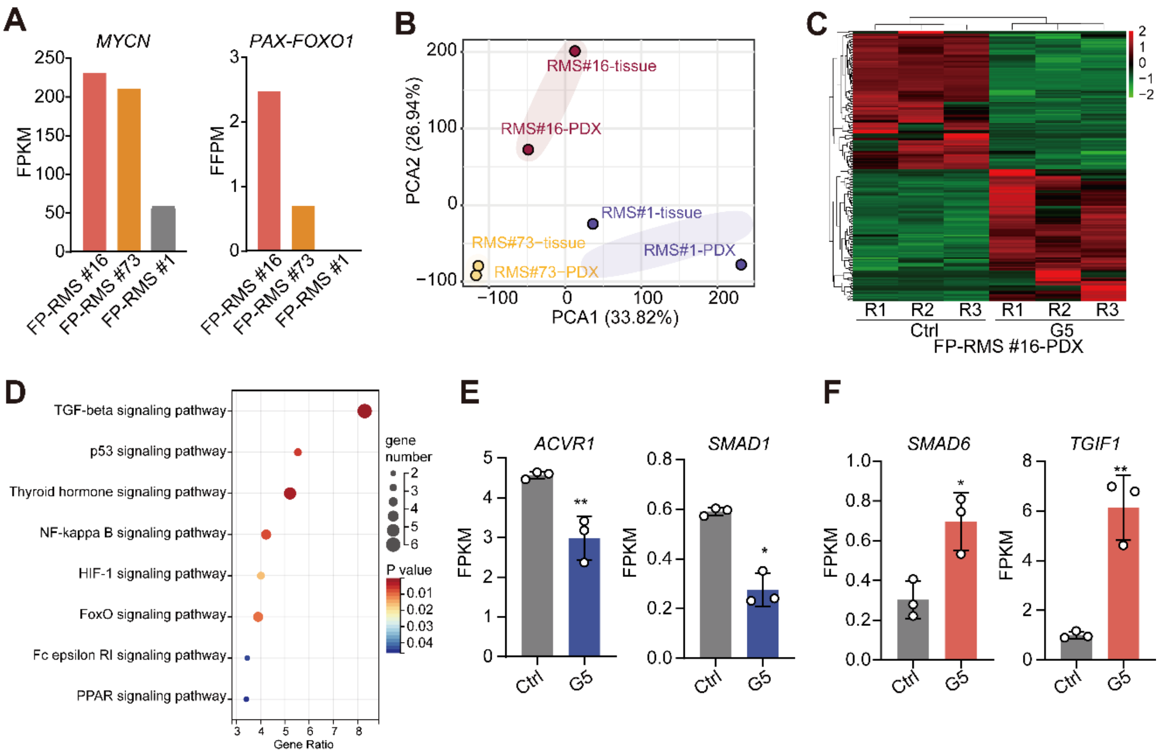

Supplementary Figure 5. Analysis of related gene levels in PDX. (A) RNA-seq analysis of MYCN and PAX3/7-FOXO1 fusion transcript expression in RMS-PDX samples. MYCN expression levels are quantified as FPKM, while the specific PAX3-FOXO1 or PAX7-FOXO1 fusion transcripts, identified by STAR-Fusion, are quantified as FFPM. **(B**), Principal component analysis comparing DNA methylation profiles of RMS PDXs, PDCs, and patient tissue. **(C**) Unsupervised hierarchical clustering was performed to compare the transcriptome profiles after G5 and the transcriptome profiles of Ctrl group. **(D**) Pathway enrichment analysis by RNA sequencing in G5-treated FP-RMS PDX models. **(E and F**), RNA sequencing analysis showed the messenger RNA changes of the TGF-beta signaling pathway. Student's *t* test. $n = 3$. $*P < 0.05$; $**P < 0.01$.

Supplementary Table 1. RNA-seq gene expression data for RD cells shown in Figure 2C.xlsx (Microsoft Excel, xlsx format)

Supplementary Table 2. Clinical Characteristics of RMS-PDC

Sample ID	Gender	Age at Surgery in years	Histological category	Subtype	Fusion type
RMS#1	female	＜10Y	RMS	ERMS	FN
RMS#10	male	＞10Y	RMS	ARMS	PAX3-FOXO1
RMS#15	male	＜10Y	RMS	ARMS	PAX3-FOXO1
RMS#16	male	＜10Y	RMS	ARMS	PAX7-FOXO1
RMS#19	female	＞10Y	RMS	ARMS	PAX3-FOXO1
RMS#24	male	＞10Y	RMS	ERMS	FN
RMS#31	male	＜10Y	RMS	ERMS	FN
RMS#32	male	＜10Y	RMS	ERMS	FN
RMS#33	male	＜10Y	RMS	unknown	FN
RMS#34	female	＜10Y	RMS	ERMS	FN
RMS#36	male	＞10Y	RMS	ERMS	FN
RMS#39	female	＞10Y	RMS	unknown	FN
RMS#40	male	＜10Y	RMS	ERMS	FN
RMS#42	female	＞10Y	RMS	unknown	FN
RMS#44	female	＜10Y	RMS	ERMS	FN

RMS#54	male	＜10Y	RMS	unknown	FN
RMS#73	female	＞10Y	RMS	ARMS	PAX3-FOXO1
RMS#75	male	＜10Y	RMS	ERMS	FN
RMS#95	female	＞10Y	RMS	ARMS	PAX3-FOXO1

Supplementary Table 3. Hairpin sequences of shRNA

	shRNA	Hairpin Sequences
1	sh*PAX3-FOXO1* #1	GGCCTCTCACCTCAGAATTCA
2	sh*PAX3-FOXO1* #2	TCTCACCTCAGAATTCAATTC
3	sh*MYCN* #1	CAGCAGCAGTTGCTAAAGAAA
4	sh*MYCN* #2	CGGACGAAGATGACTTCTACT
5	sh*YOD1* #1	GAGTACTGTGACTGGATCAAA
6	sh*YOD1* #2	CCAGAAGTTCACCTGCATTTA

Supplementary Table 4. Sequences of RT-PCR primers

	Primers	5'-3' sequences
1	*PAX3-FOXO1*-F	CCTCTCACCTCAGAATTCAATT
	PAX3-FOXO1-R	TCTGGATTGAGCATCCACCAAG
2	*PAX7-FOXO1*-F	AGCTTCTCCAGCTACTCTGAC
	PAX7-FOXO1-R	CTCTGGATTGAGCATCCACC
3	*MYCN*-F	ACCACAAGGCCCTCAGTACC
	MYCN-R	TCTCCACAGTGACCACGTCGATTT

	Primers	5'-3' sequences
4	*GAPDH*-F	CTGGGCTACACTGAGCACC
	GAPDH-R	AAGTGGTCGTTGAGGGCAATG
5	*YOD1*-F	CAGCGTAACTTCCCTGATCCA
	YOD1-R	TGGCATGTTCCCTTGCTTC

Supplementary Table 5. Sequences of sgRNA

sgRNA	Clone Sequence
sgRNA#1	Sense (5' to 3'): CACCGATCACCGGGATCGCCCCCGG
	Antisense (5' to 3'): AAACCCGGGGGCGATCCCGGTGATC
sgRNA#2	Sense (5' to 3'): CACCGGCGGTCAGCGAATCCTCGT
	Antisense (5' to 3'): AAACACGAGGATTCGCTGACCGCC
