# Supplementary material for: Therapeutic targeting of YOD1 disrupts the PAX-FOXO1/N-Myc feedback loop in rhabdomyosarcoma
Source: JCI Insight. 2025 Dec 16;11(3):e193221. doi: 10.1172/jci.insight.193221 (PMC12892920; doi:10.1172/jci.insight.193221)

Unedited blot and gel images

Fig 1B

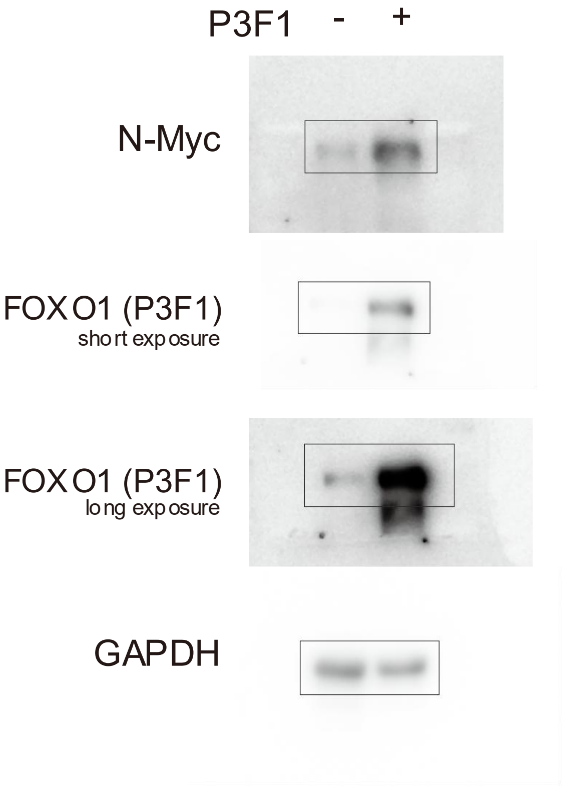

Fig 1D

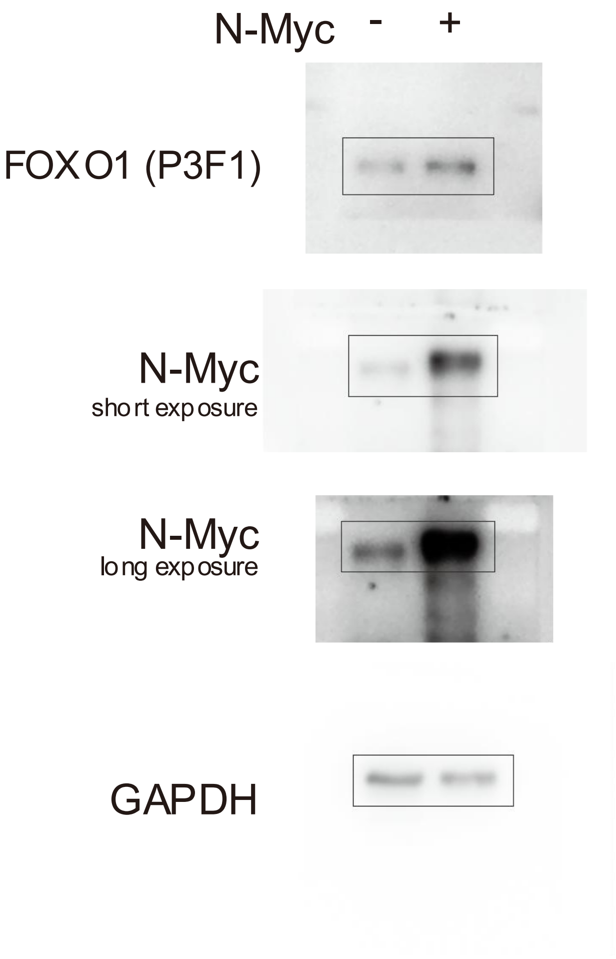
N-Myc
-
+
FOXO1 (P3F1)
N-Myc
short exposure
N-Myc
long exposure
GAPDH

Fig 1F

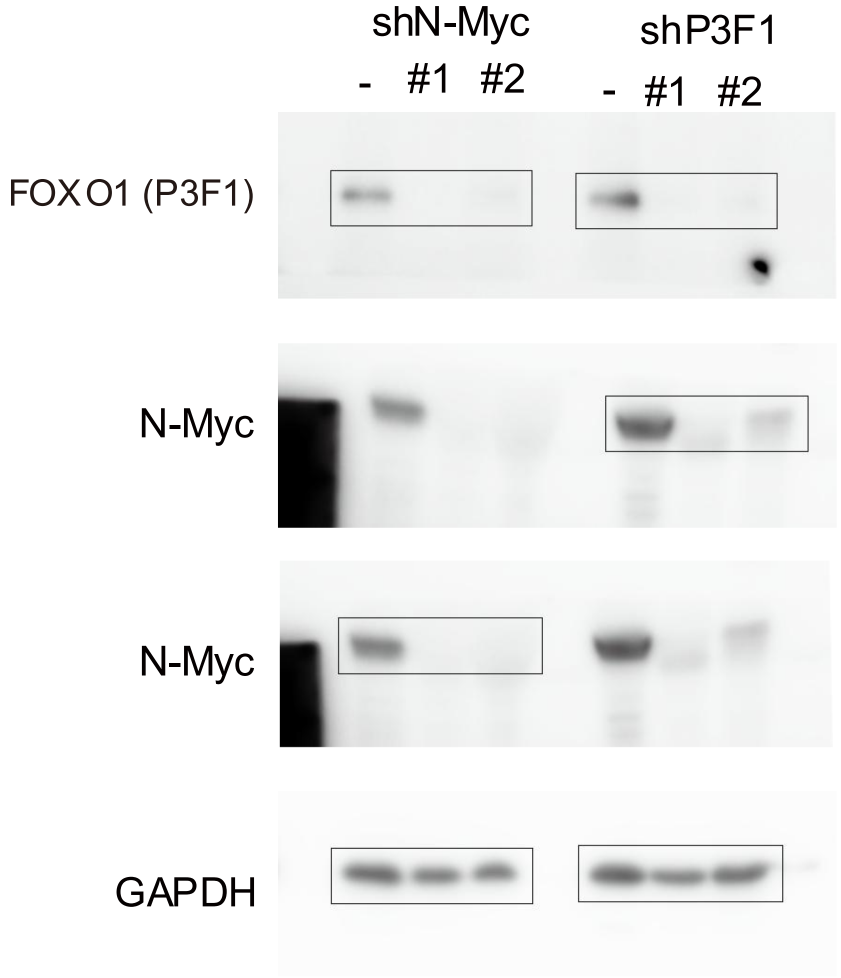

Fig 2D

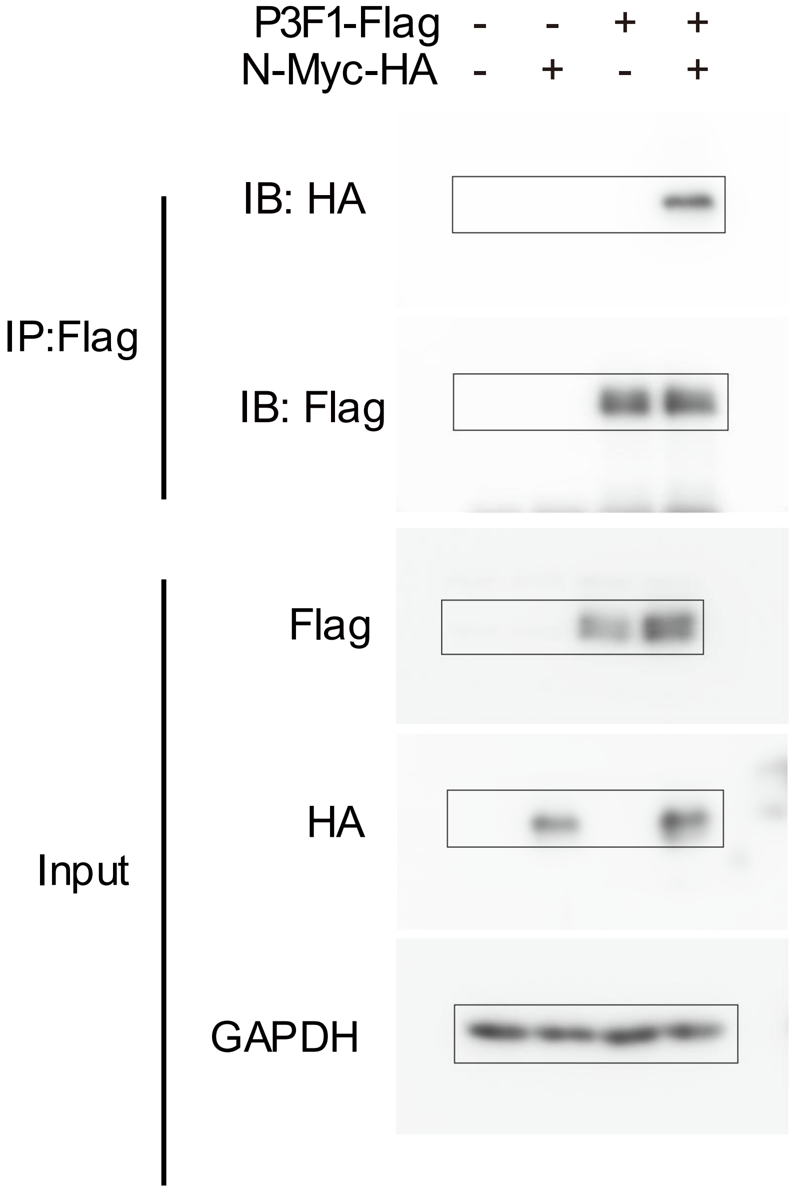

Fig 2E

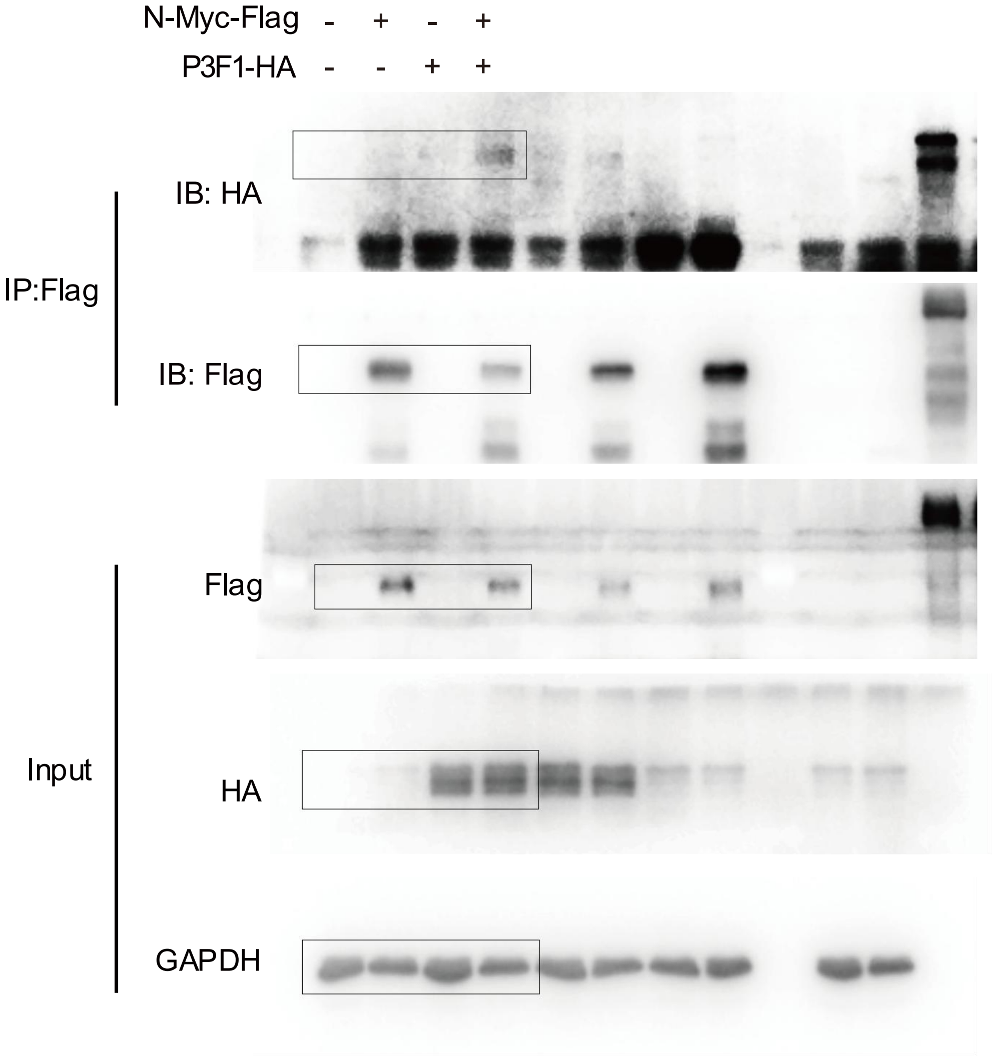

Fig 2F

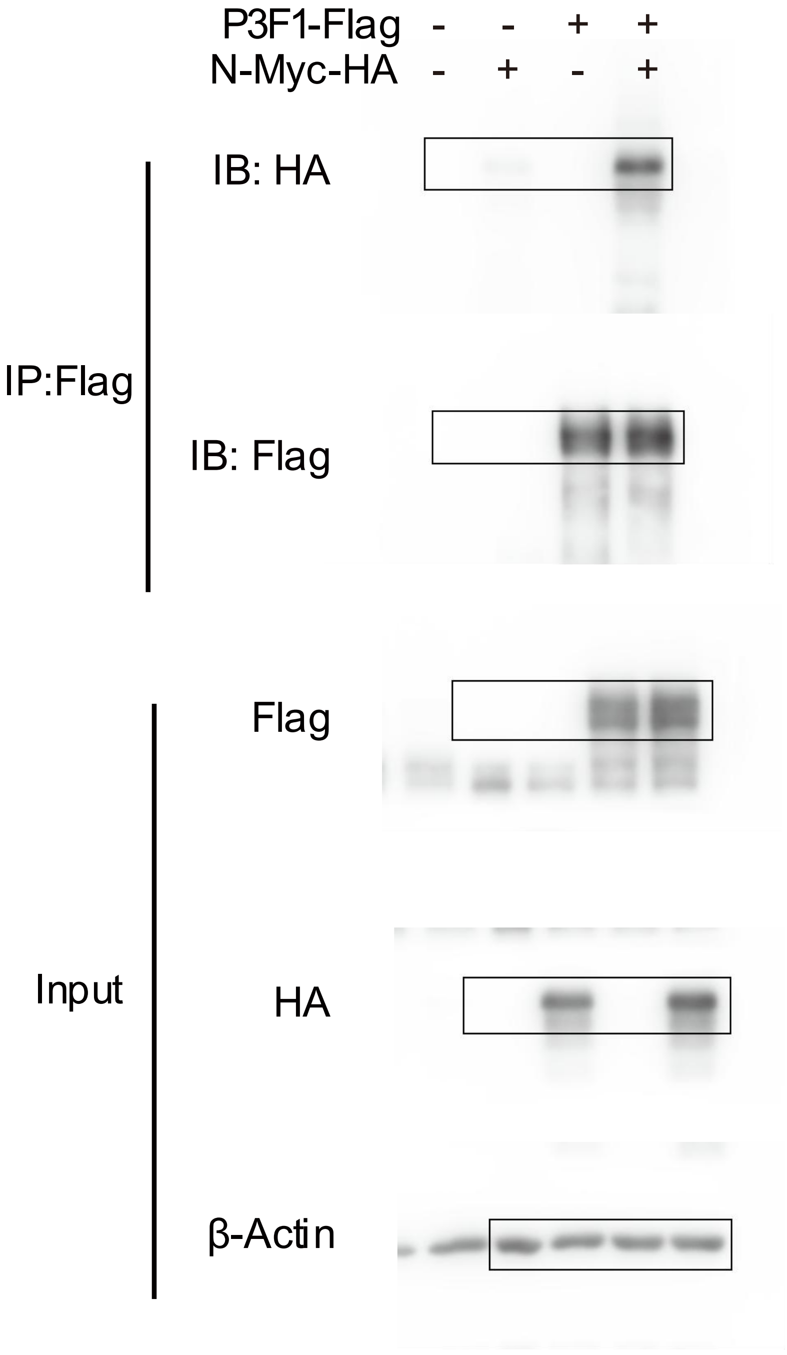

Fig 3C

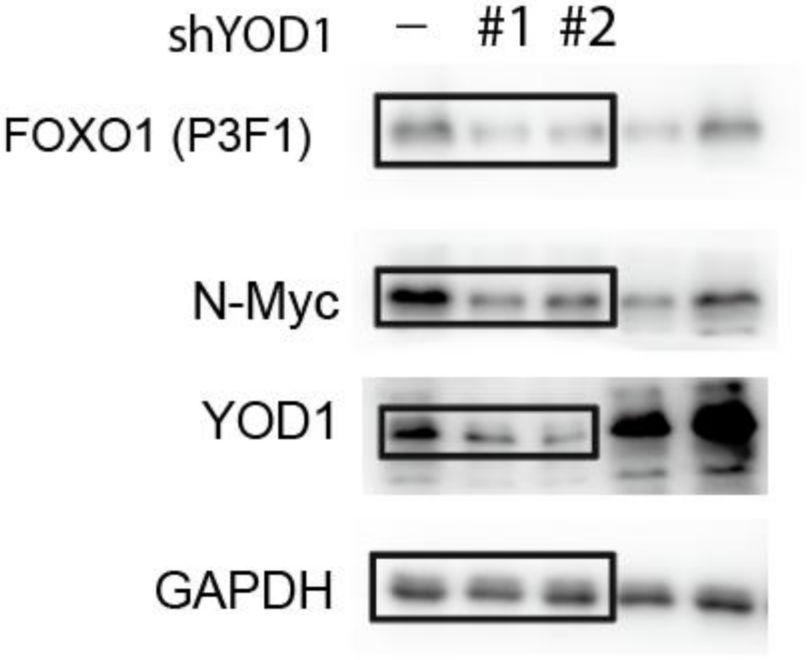

Fig 3D

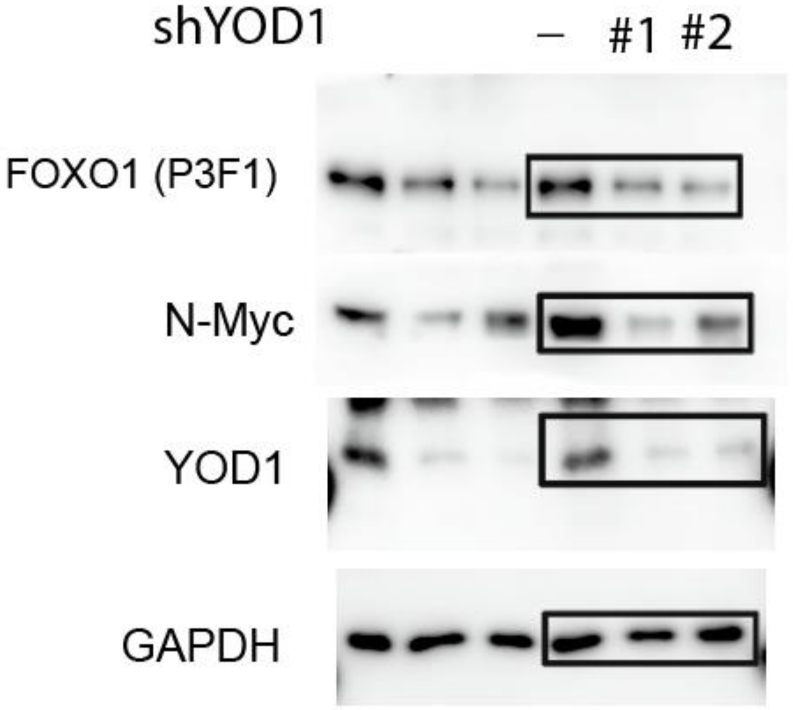

Fig 3E

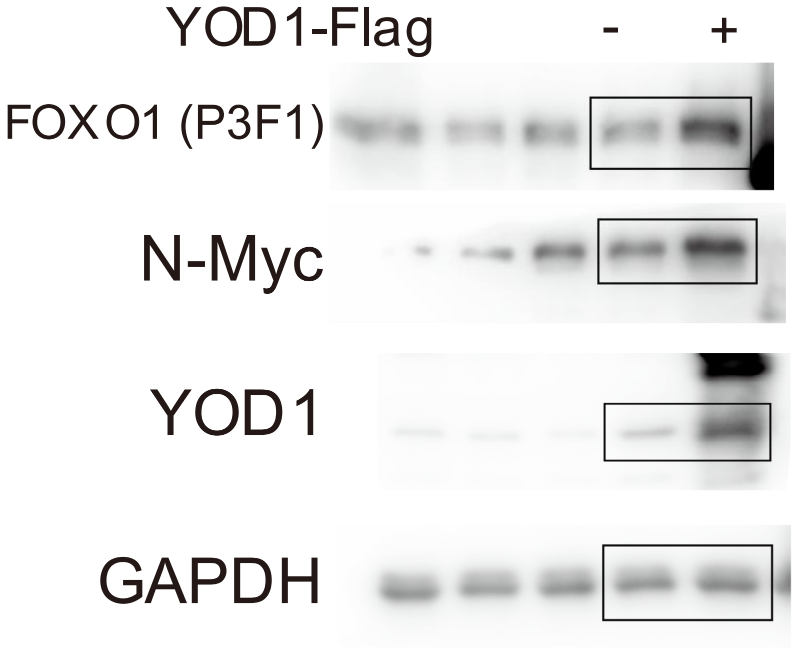

Fig 3F

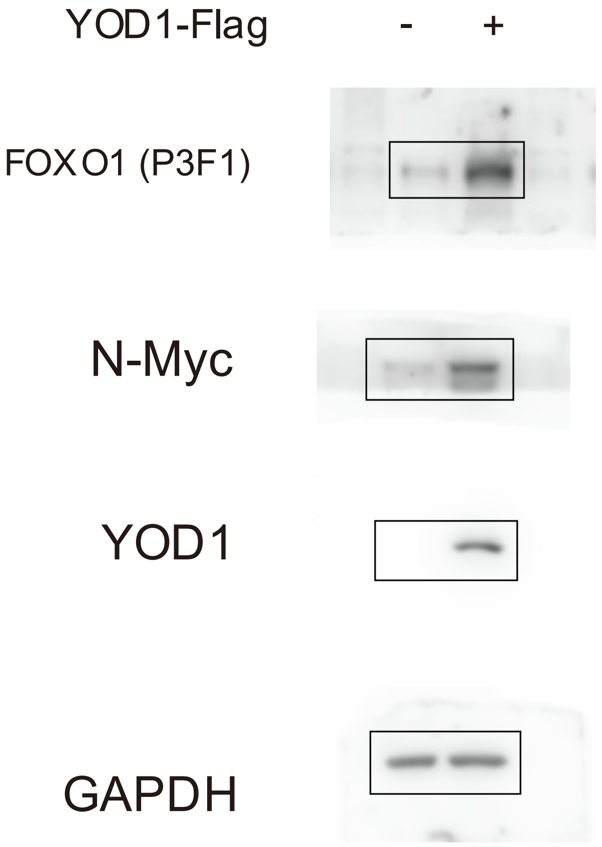

Fig 4 A

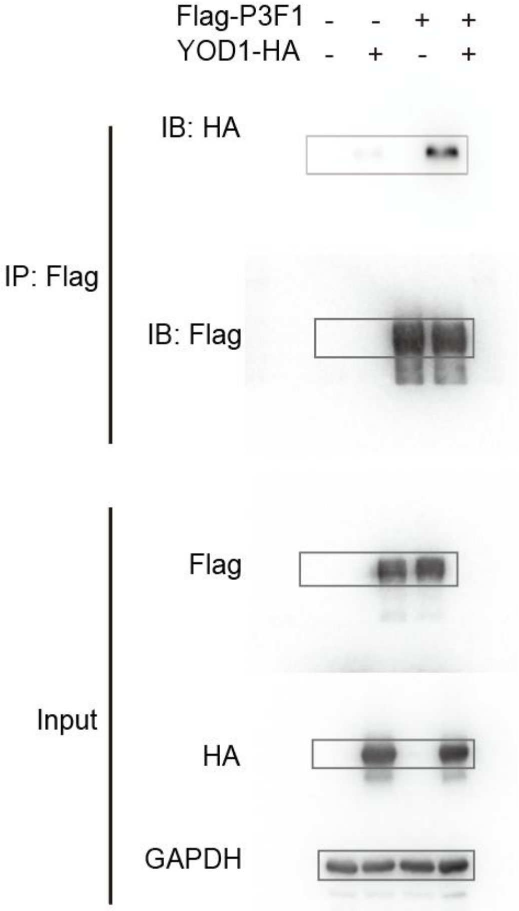
Flag-P3F1 - - + +
YOD1-HA - + - +
IB: HA
IP: Flag
IB: Flag
Flag
Input
HA
GAPDH

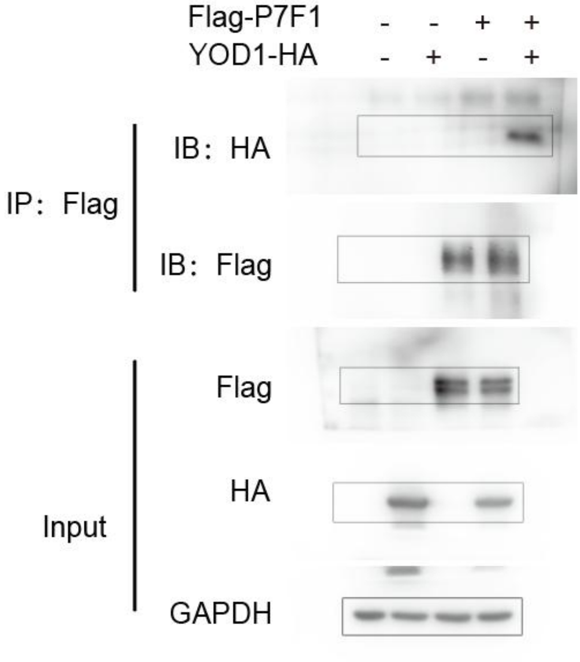
Flag-P7F1 - - + +
YOD1-HA - + - +
IB：HA
IP：Flag
IB：Flag
Flag
HA
Input
GAPDH

Fig 4 B

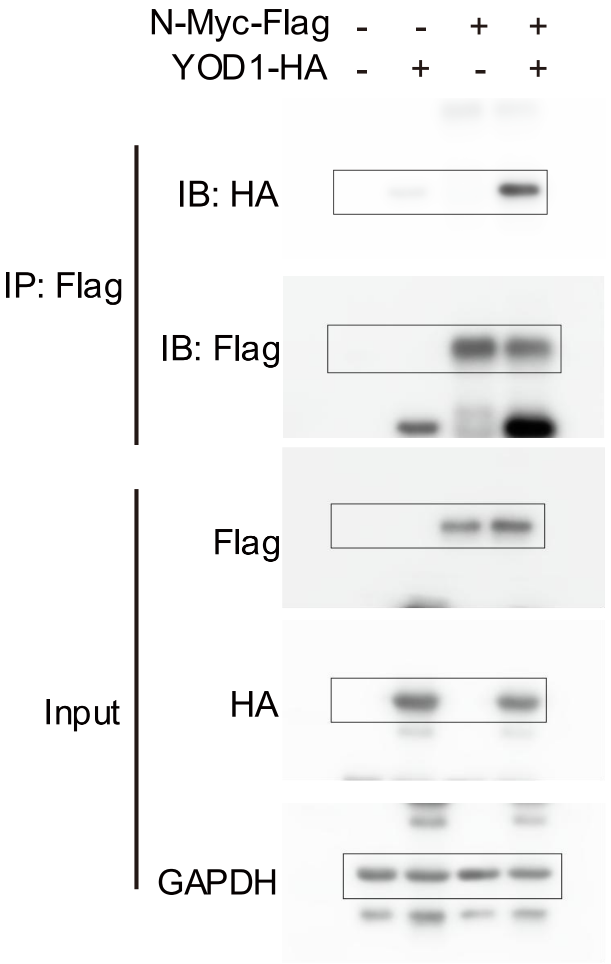

Fig 4 C

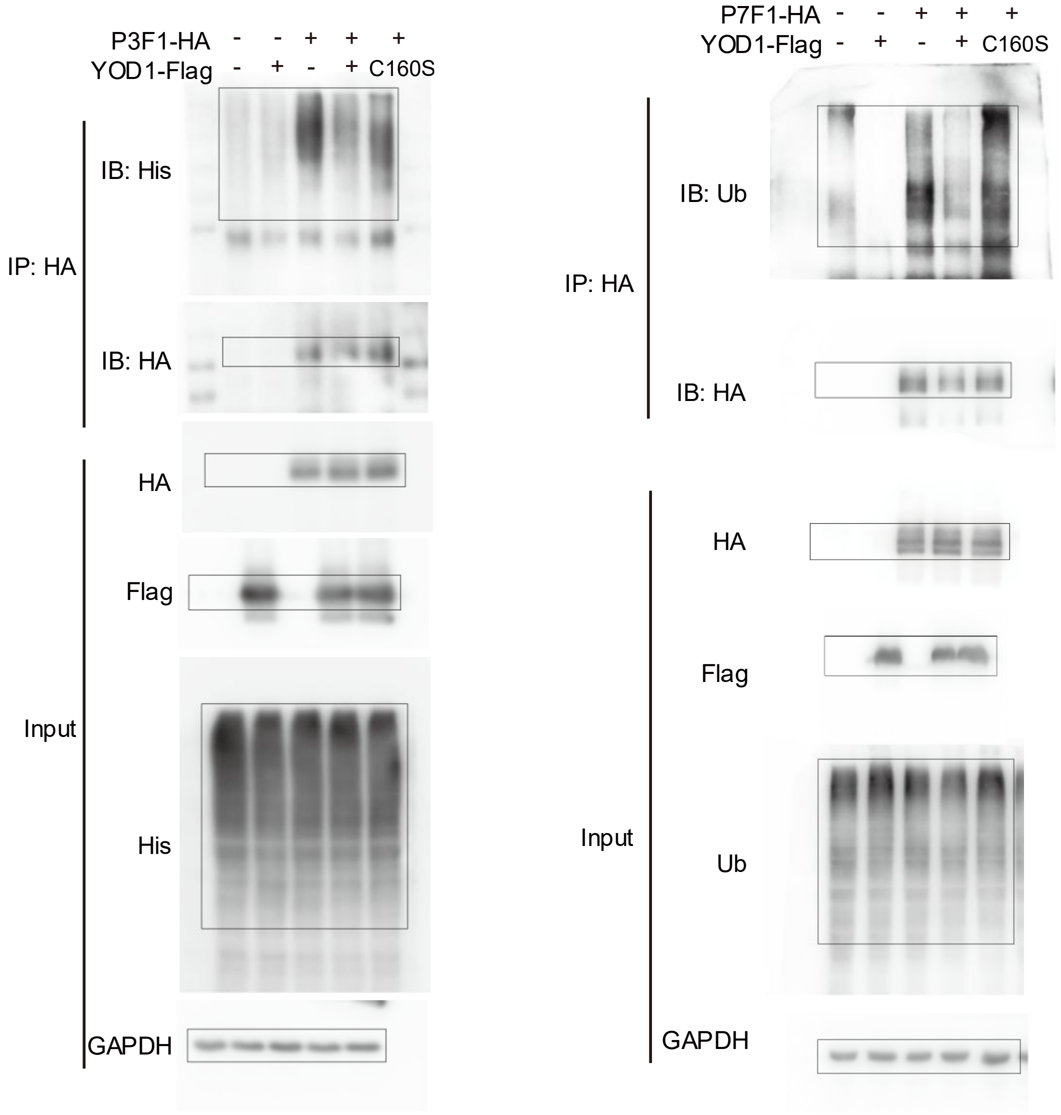
P3F1-HA - - + + +
YOD1-Flag - + - + C160S
IB: His
IP: HA
IB: HA
HA
Flag
Input
His
GAPDH
P7F1-HA - - + + +
YOD1-Flag - + - + C160S
IB: Ub
IP: HA
IB: HA
HA
Flag
Input
Ub
GAPDH

Fig 4 D

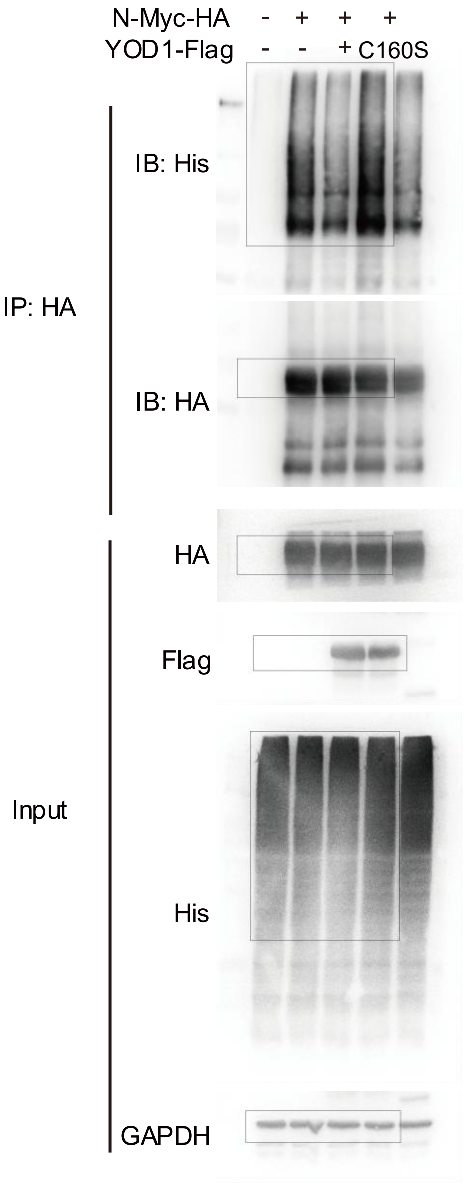

N-Myc-HA - + + +
YOD1-Flag - - + C160S
IP: HA
IB: His
IB: HA
Input
HA
Flag
His
GAPDH

Fig 5 B

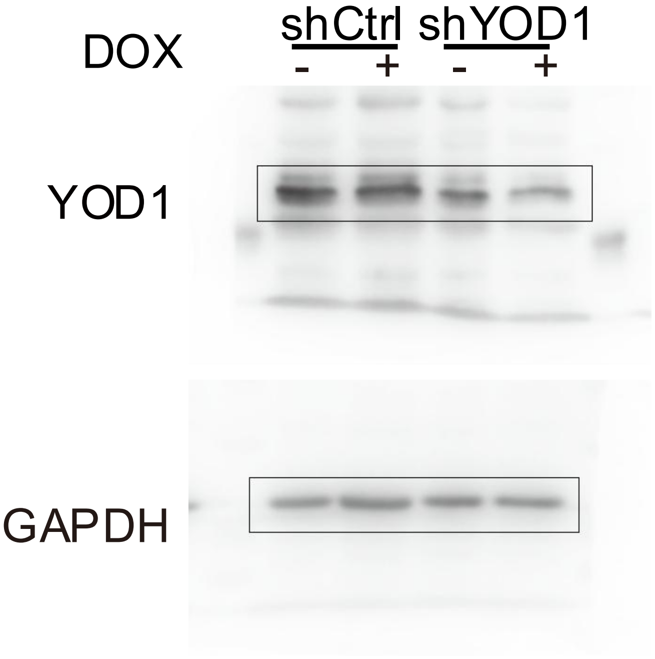

Fig 5E

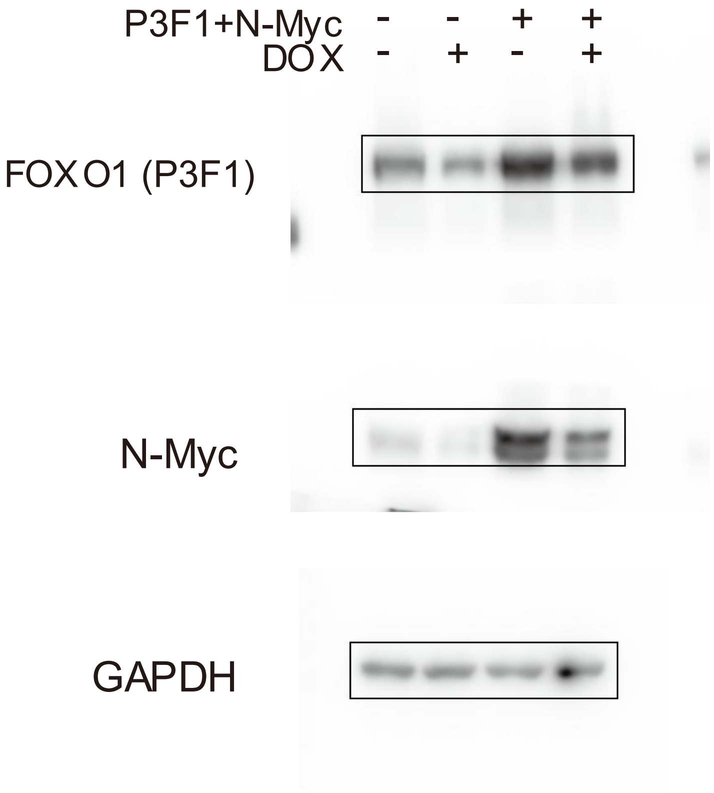

Fig 6C

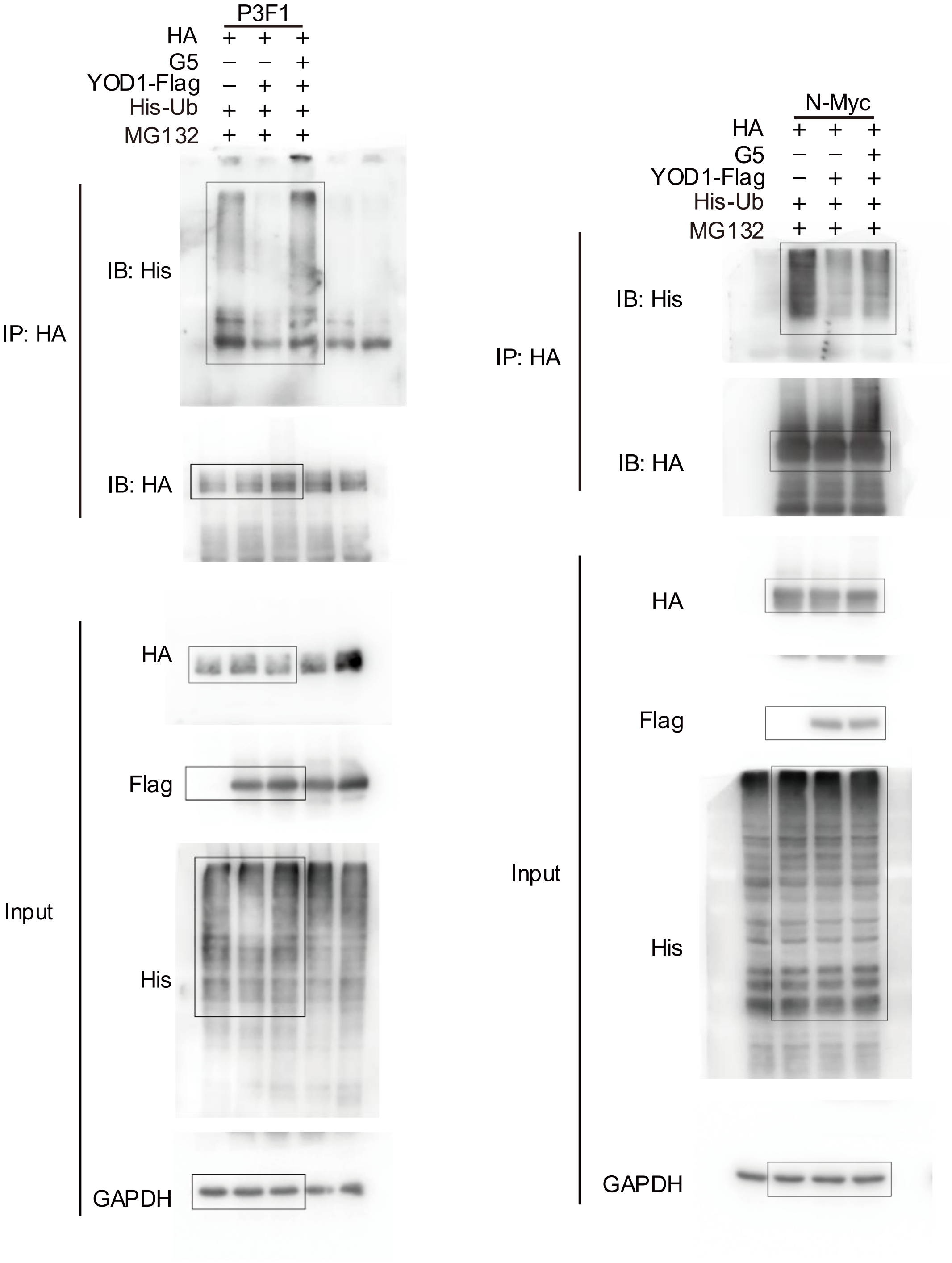
P3F1
HA + + +
G5 − − +
YOD1-Flag − + +
His-Ub + + +
MG132 + + +
IP: HA
IB: His
IB: HA
Input
HA
Flag
His
GAPDH
N-Myc
HA + + +
G5 − − +
YOD1-Flag − + +
His-Ub + + +
MG132 + + +
IP: HA
IB: His
IB: HA
Input
HA
Flag
His
GAPDH

Fig 6D

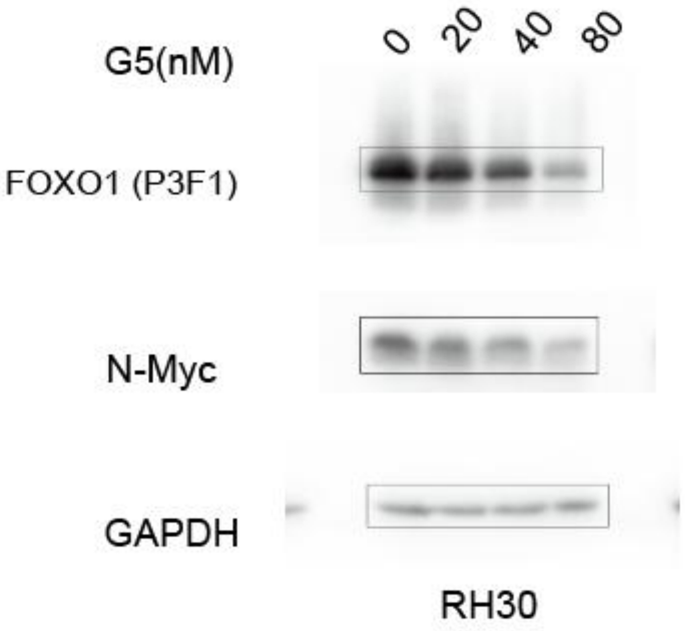

Fig 6E

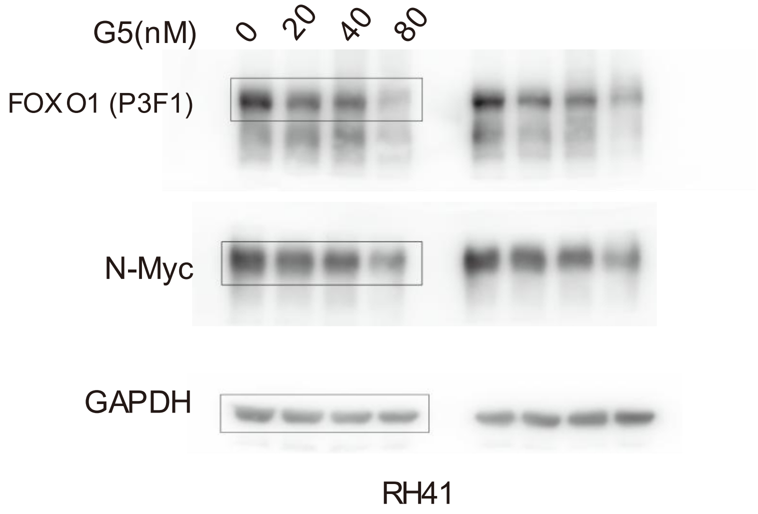

Fig 6 I

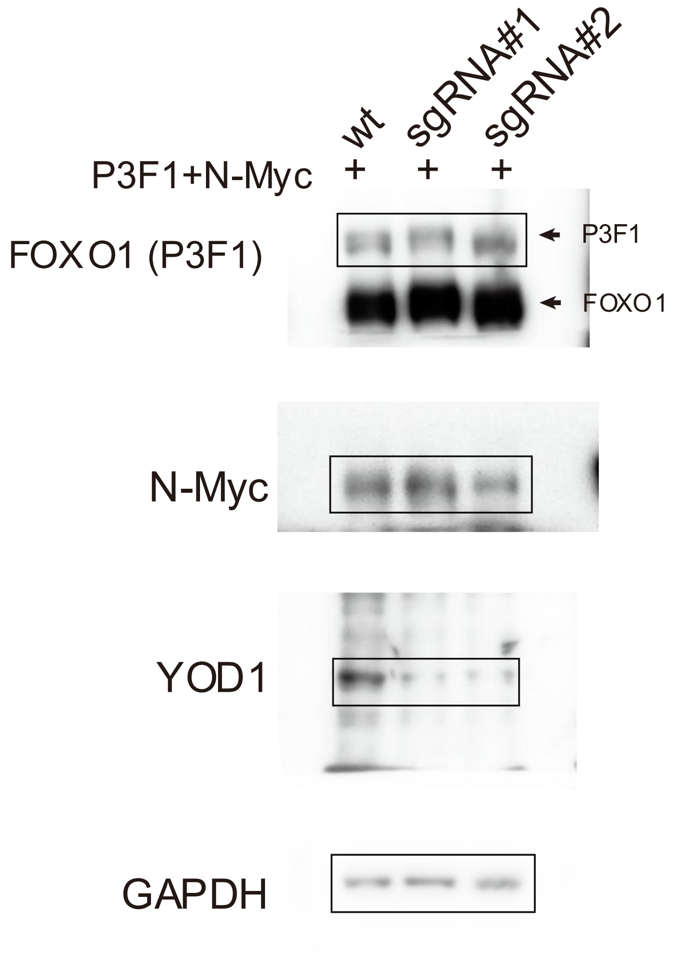
wt
sgRNA#1
sgRNA#2
P3F1+N-Myc
+
+
+
FOXO1 (P3F1)
P3F1
FOXO1
N-Myc
YOD1
GAPDH

Fig 7E

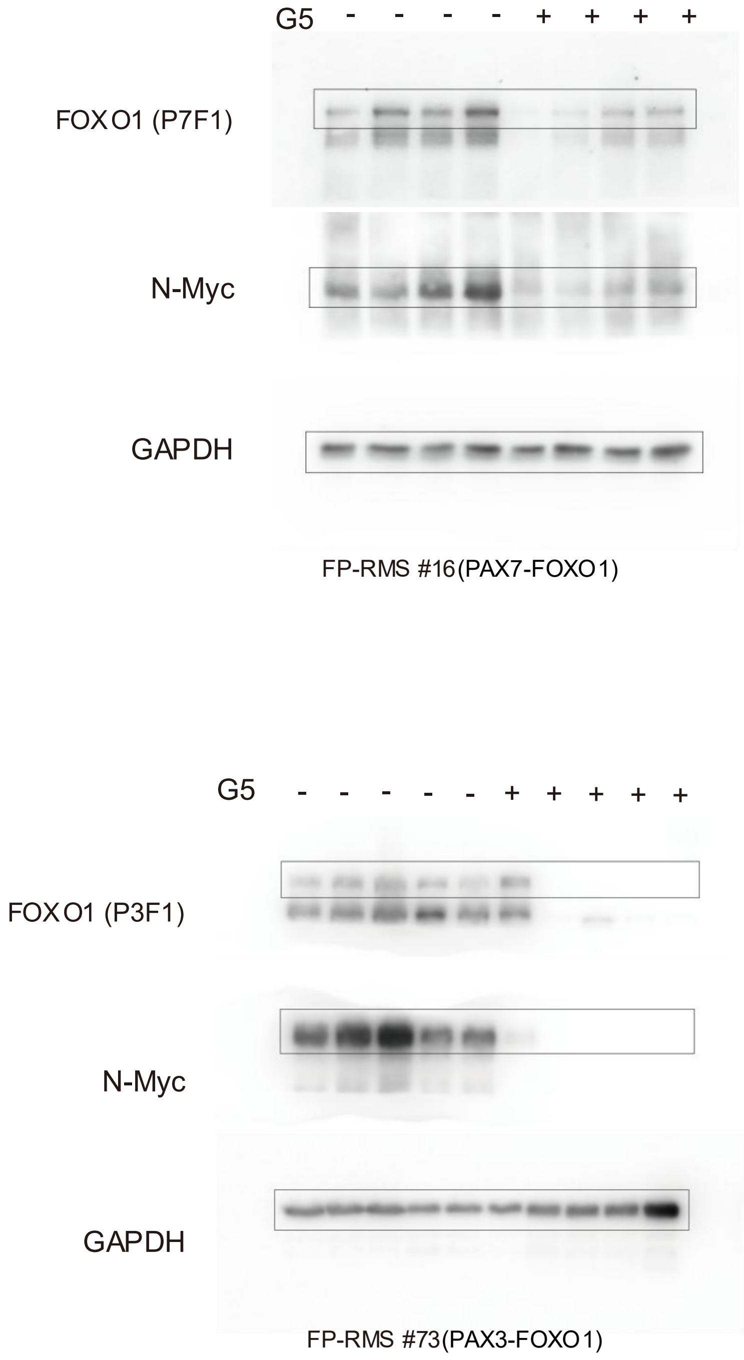

G5 - - - - + + + +
FOXO1 (P7F1)
N-Myc
GAPDH
FP-RMS #16(PAX7-FOXO1)
G5 - - - - - + + + + +
FOXO1 (P3F1)
N-Myc
GAPDH
FP-RMS #73(PAX3-FOXO1)

Fig S1A

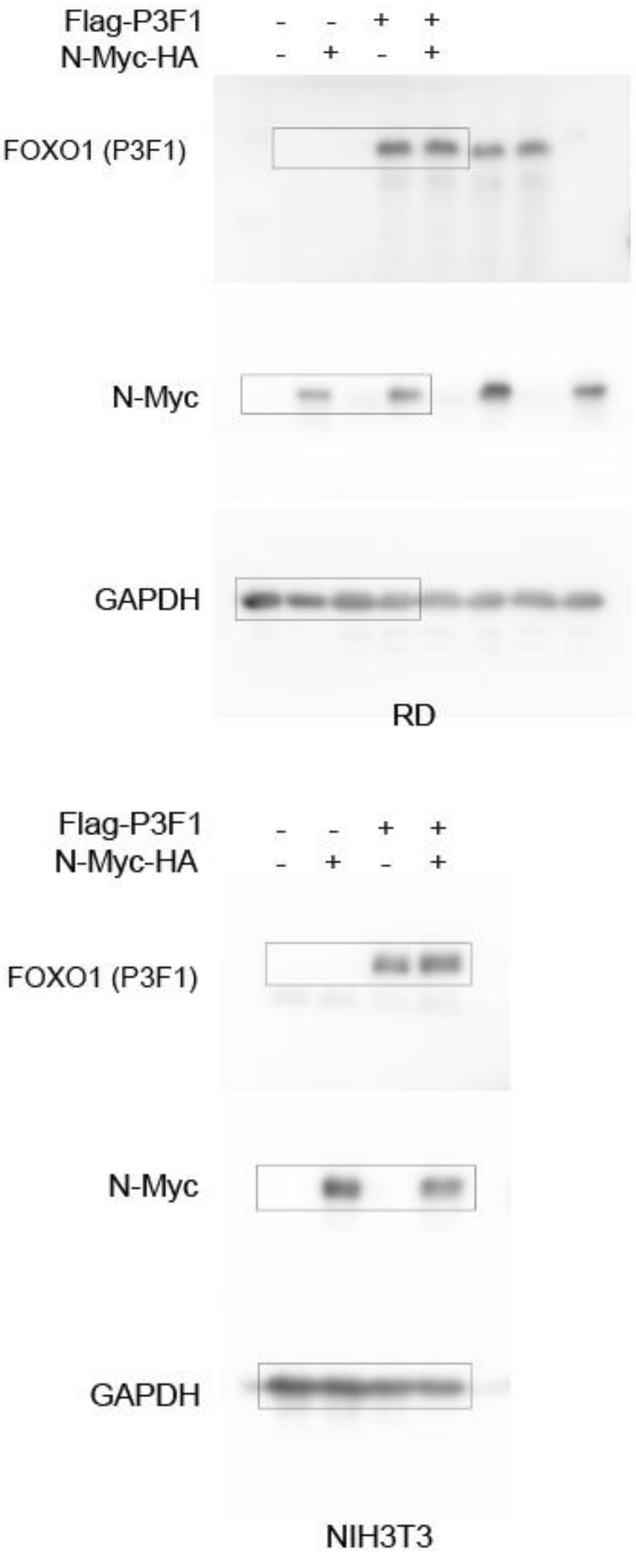
Flag-P3F1 - - + +
N-Myc-HA - + - +
FOXO1 (P3F1)
N-Myc
GAPDH
RD
Flag-P3F1 - - + +
N-Myc-HA - + - +
FOXO1 (P3F1)
N-Myc
GAPDH
NIH3T3

Fig S1B

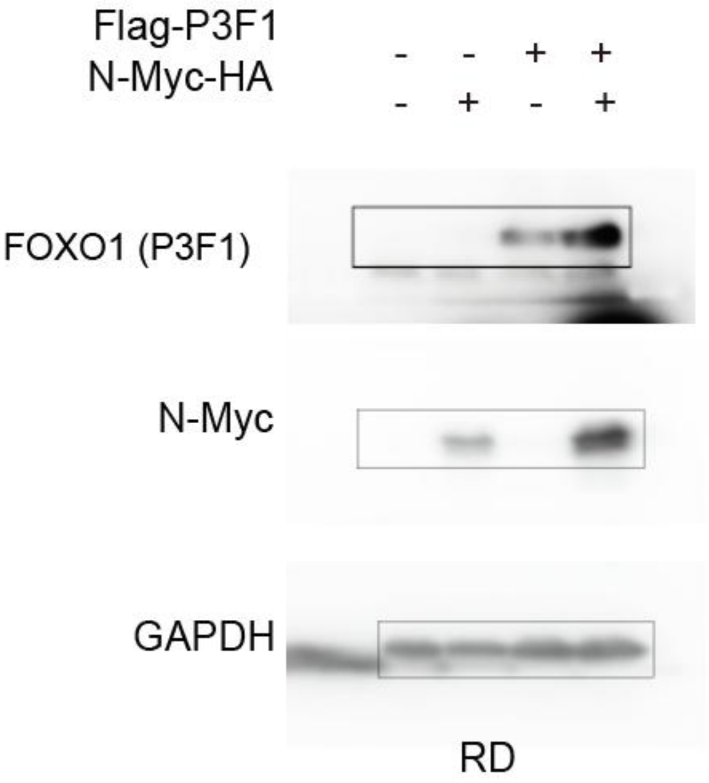

Flag-P3F1
N-Myc-HA
- - + +
- + - +
FOXO1 (P3F1)
N-Myc
GAPDH
RD

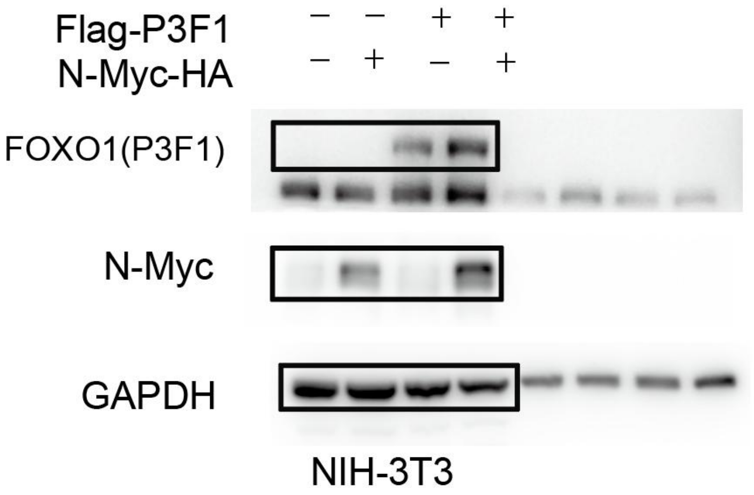

Flag-P3F1
N-Myc-HA
– – + +
– + – +
FOXO1(P3F1)
N-Myc
GAPDH
NIH-3T3

Fig S2C

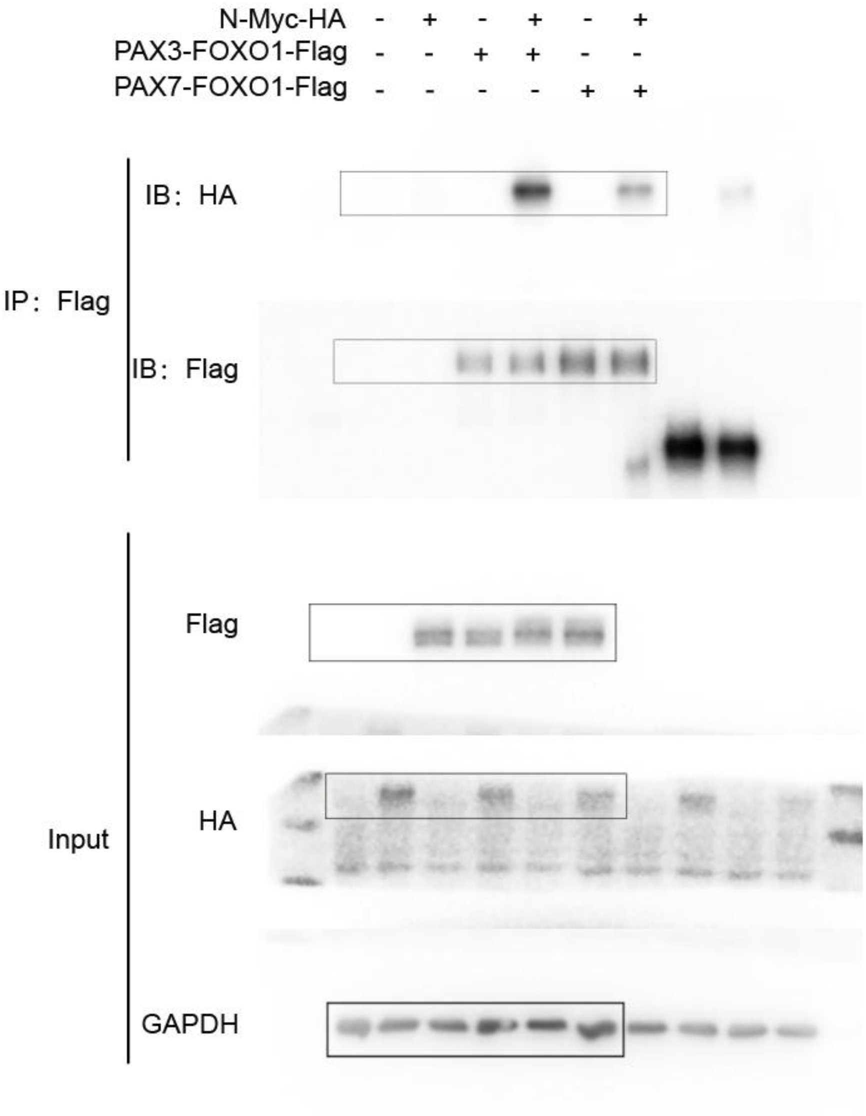

N-Myc-HA - + - + - +
PAX3-FOXO1-Flag - - + + - -
PAX7-FOXO1-Flag - - - - + +
IB：HA
IP：Flag
IB：Flag
Flag
Input
HA
GAPDH

Fig S2D

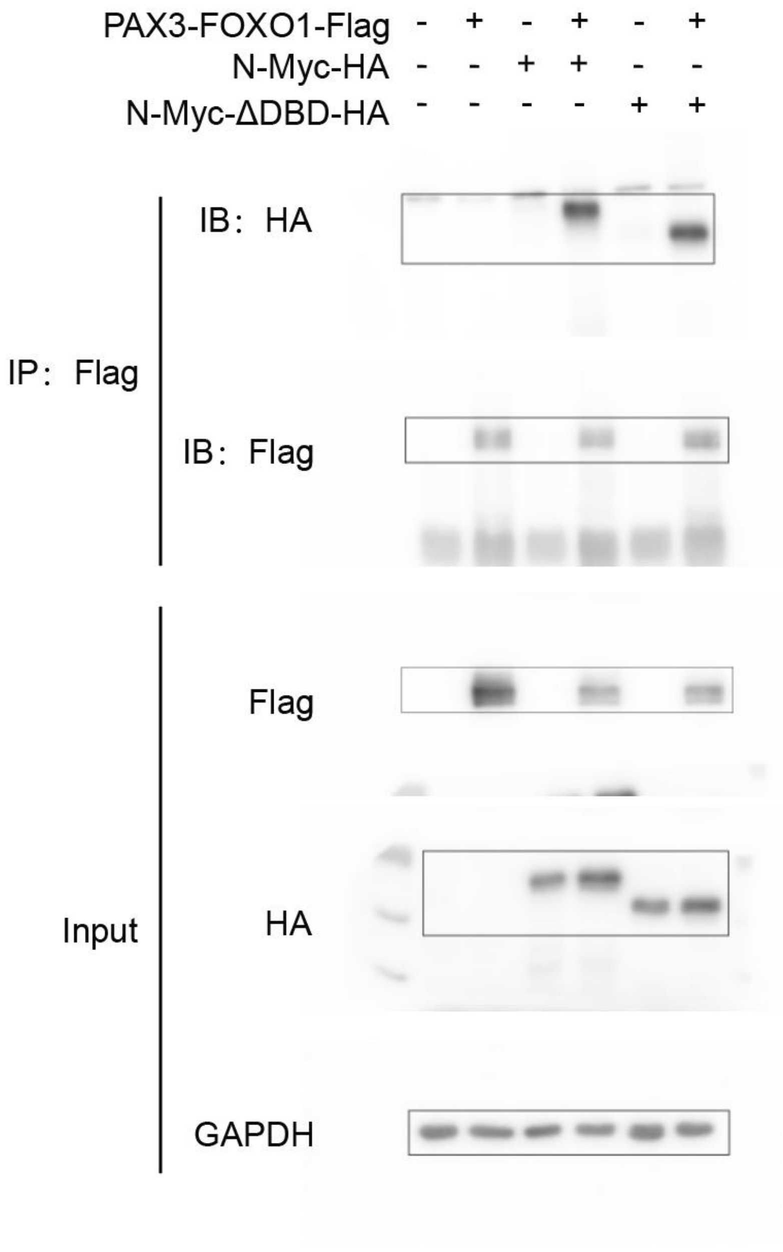

PAX3-FOXO1-Flag - + - + - +
N-Myc-HA - - + + - -
N-Myc-ΔDBD-HA - - - - + +
IP：Flag
IB：HA
IB：Flag
Input
Flag
HA
GAPDH

Fig S3A

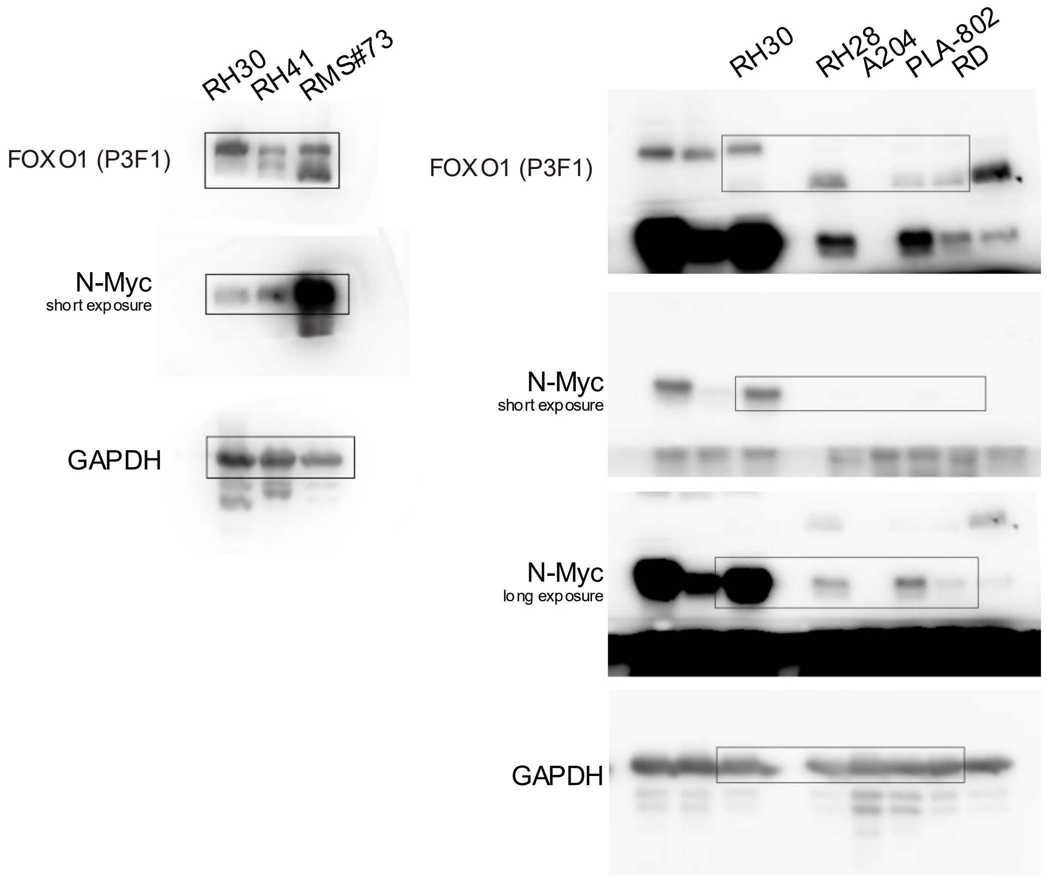
RH30
RH41
RMS#73
FOXO1 (P3F1)
N-Myc
short exposure
GAPDH
RH30
RH28
A204
PLA-802
RD
FOXO1 (P3F1)
N-Myc
short exposure
N-Myc
long exposure
GAPDH

Fig S3B

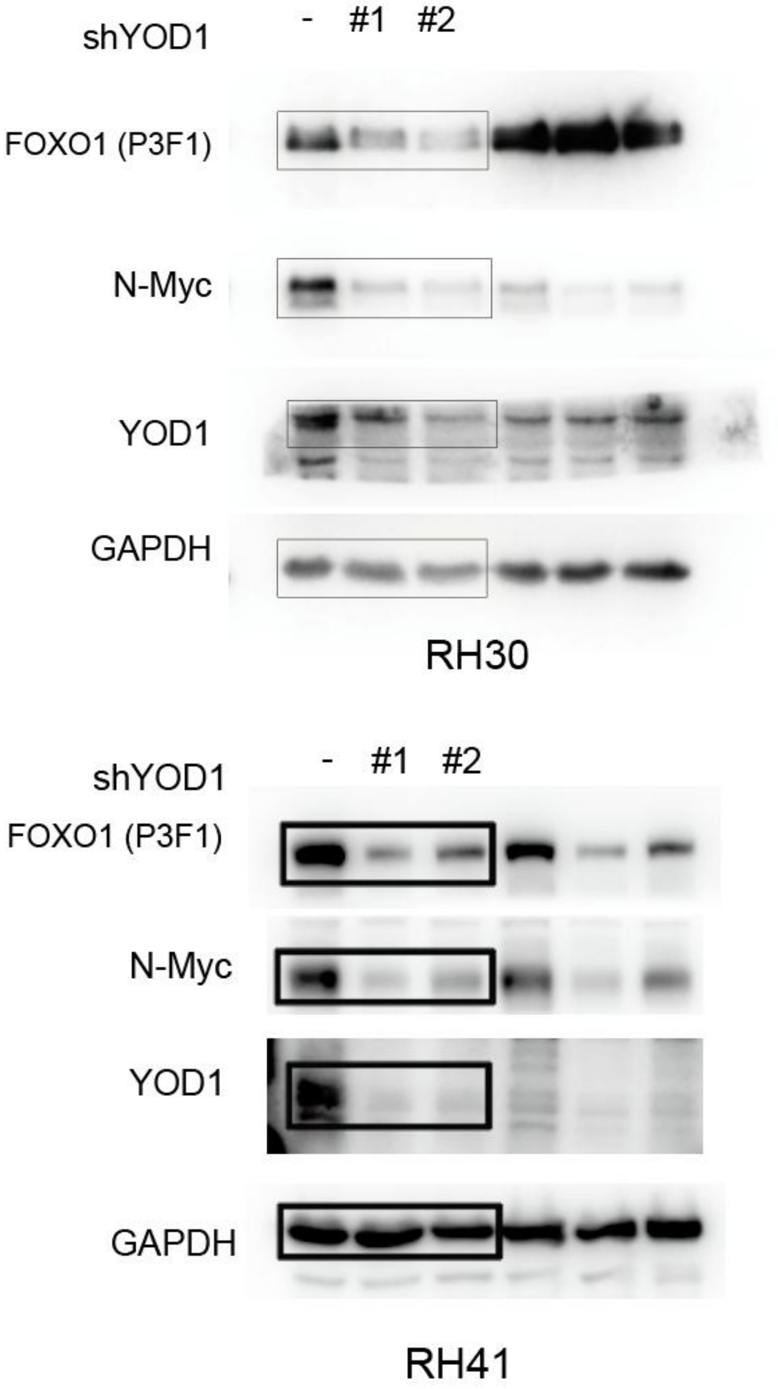
shYOD1
- #1 #2
FOXO1 (P3F1)
N-Myc
YOD1
GAPDH
RH30
shYOD1
- #1 #2
FOXO1 (P3F1)
N-Myc
YOD1
GAPDH
RH41

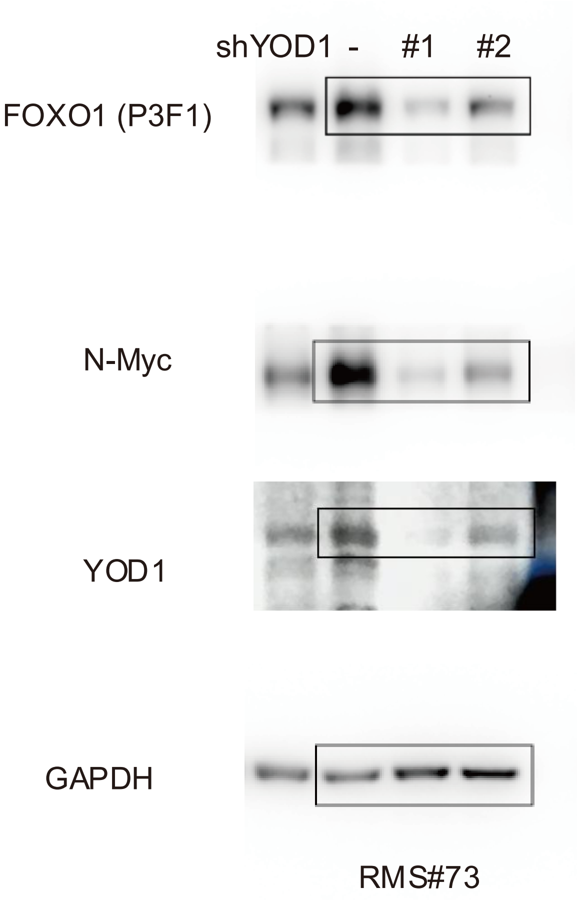
shYOD1 - #1 #2
FOXO1 (P3F1)
N-Myc
YOD1
GAPDH
RMS#73

Fig S3C

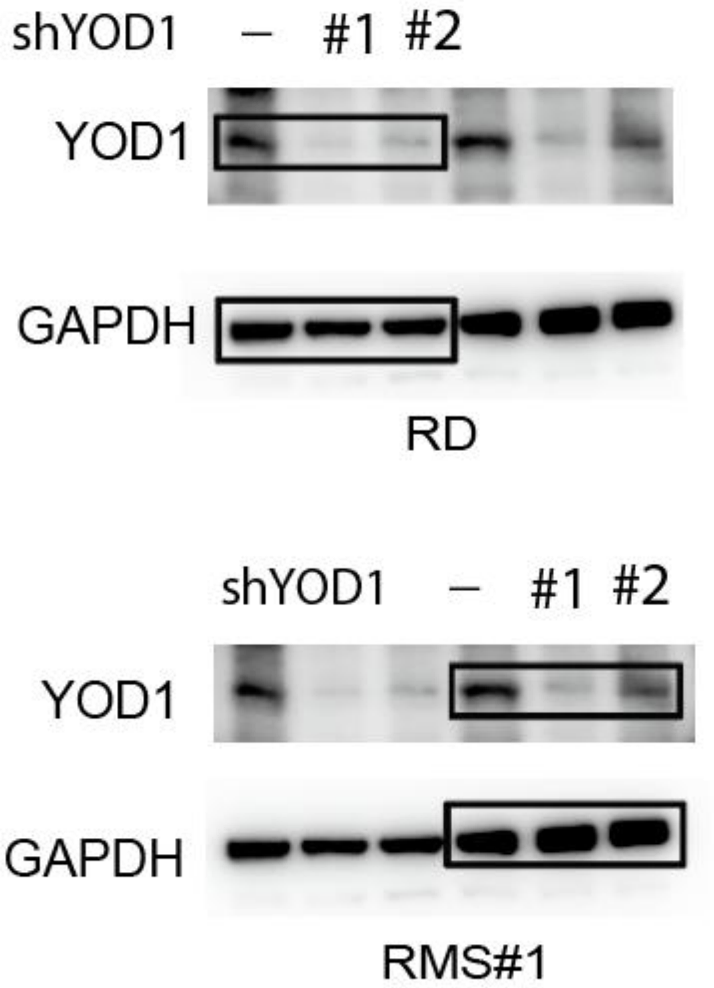

Fig S3D

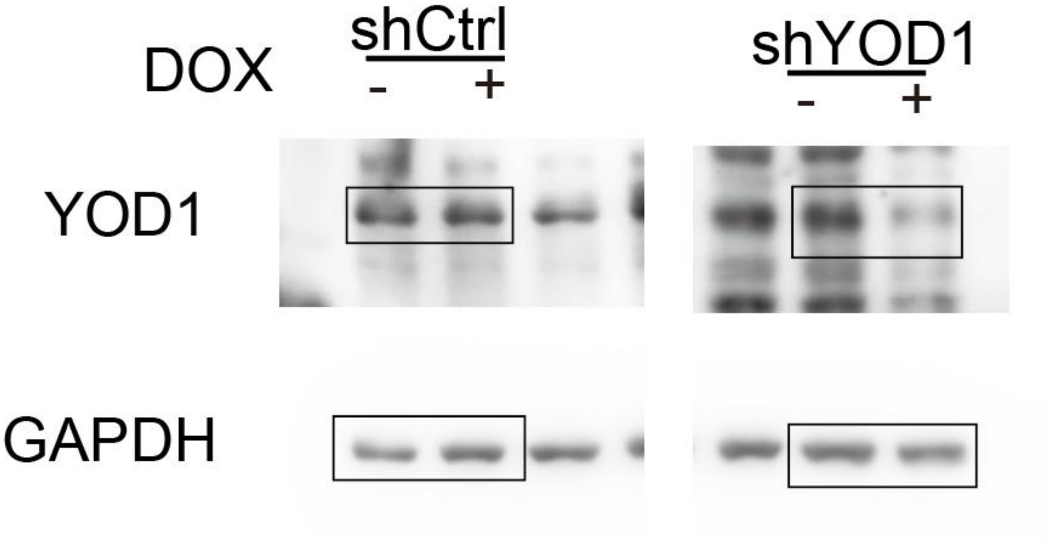

Supplement: Unedited blot and gel images [file jciinsight-11-193221-s061.pdf]
